# Confocal laser endomicroscopy for upper gastrointestinal neoplasia: Systematic review and meta-analysis

**DOI:** 10.1055/a-2863-1407

**Published:** 2026-05-12

**Authors:** Ji-Yeon Lee, Seung-Hee Lee, Young-Geon Ji, Hwoon-Yong Jung, Ja-Seong Koo, Jae-Myung Park

**Affiliations:** 1 Division of Health Technology Assessment Research 205612 National Evidence-Based Healthcare Collaborating AgencyGwangjin-guSeoulKorea (the Republic of); 2 Preventive Medicine 451070 CHA University School of MedicinePocheon-siGyeonggi-doKorea (the Republic of); 3 Division of Gastroenterology, Asan Medical Center 37994 University of Ulsan College of MedicineSongpa-guSeoulKorea (the Republic of); 4 Pathology 37991 Yonsei University College of MedicineSeodaemun-guSeoulKorea (the Republic of); 5 Department of Internal Medicine, Seoul St. Mary's Hospital 37128 The Catholic University of Korea College of MedicineSeocho-guSeoulKorea (the Republic of)

**Keywords:** Endoscopy Upper GI Tract, Barrett's and adenocarcinoma, Precancerous conditions & cancerous lesions (displasia and cancer) stomach, Quality and logistical aspects, Performance and complications

## Abstract

**Background and study aims:**

Probe-based confocal laser endomicroscopy (pCLE) enables real-time, in vivo microscopic evaluation of upper gastrointestinal mucosa. Although many studies have evaluated its diagnostic performance, comprehensive synthesis of its accuracy, safety, and clinical utility remains limited.

**Methods:**

We performed a systematic review and meta-analysis of studies assessing diagnostic accuracy, safety, and clinical utility of pCLE for detecting dysplasia or neoplasia in the upper gastrointestinal tract. MEDLINE, Embase, and the Cochrane Library were searched from inception to October 2024 for studies comparing pCLE with histopathology. Study quality was assessed using QUADAS-2. Pooled sensitivity and specificity were estimated using a bivariate random-effects model. Safety outcomes and clinical impact were narratively summarized.

**Results:**

Thirty-three studies (n = 2,350 patients) were included: 15 focused on esophageal, 16 on gastric, and two on mixed upper gastrointestinal lesions. For esophageal neoplasia, pooled sensitivity was 0.89 (95% confidence interval [CI] 0.84–0.92) and specificity was 0.79 (95% CI 0.68–0.87). For gastric neoplasia, pooled sensitivity was 0.89 (95% CI 0.83–0.93) and specificity was 0.95 (95% CI 0.92–0.96). In selected clinical settings, such as Barrett's esophagus with subtle mucosal and no discrete macroscopic lesions, adjunctive pCLE altered real-time therapeutic management in up to 69.2% of cases (9/13). Only minor adverse events, such as transient bleeding or fluorescein-related reactions, were reported.

**Conclusions:**

pCLE is a safe and accurate adjunct to standard upper gastrointestinal endoscopy, particularly when lesion conspicuity is low or histologic confirmation is important. However, pooled estimates, particularly the high specificity for gastric lesions, should be interpreted cautiously due to potential small-study effects.

## Introduction

Upper gastrointestinal neoplasia, encompassing conditions such as Barrett’s esophagus (BE), early gastric cancer, and high-grade dysplasia (HGD), remains a significant global health concern, contributing substantially to cancer-related morbidity and mortality worldwide. Early detection and accurate characterization of neoplastic lesions are essential for guiding timely and effective intervention. Although standard white-light endoscopy (WLE), often augmented by advanced imaging modalities such as narrow-band imaging (NBI) or magnification, has improved lesion visualization, it frequently necessitates random or targeted biopsies and remains subject to interobserver variability 1 2 . This conventional approach can lead to unnecessary invasive procedures and delays in definitive diagnosis and treatment.

Probe-based confocal laser endomicroscopy (pCLE) enables real-time, in vivo histological evaluation of gastrointestinal mucosa at subcellular resolution during endoscopy. Utilizing intravenous fluorescein, pCLE visualizes microvascular and glandular structures, offering optical biopsies that enhance lesion targeting, minimize unnecessary sampling, and support immediate therapeutic decisions. This technology is especially beneficial for flat, multifocal, or ambiguous lesions, improving patient experience by reducing biopsy procedures and facilitating prompt treatment, essential objectives in contemporary patient-centered endoscopy.

Despite its theoretical advantages and promising early results, integration of pCLE into routine clinical practice remains limited. Current guidelines from the European Society of Gastrointestinal Endoscopy and American Society for Gastrointestinal Endoscopy acknowledge its potential but stop short of recommending widespread adoption 3 4 . Key barriers include high cost of equipment, limited availability of trained operators, and a lack of large-scale comparative evidence demonstrating clinical benefit and cost effectiveness. Furthermore, application of pCLE in real-world settings is inconsistent and largely restricted to expert centers.

Given this background, a systematic and comprehensive review of the current literature is critically warranted, not only to quantify diagnostic performance and safety profile, but also to clarify real-world clinical impact of pCLE. Specifically, this review sought to determine how pCLE contributes to reducing biopsy burden, guiding more precise interventions, and potentially improving patient outcome.

## Methods

### Design

This systematic review was conducted in accordance with the Cochrane Handbook for Systematic Reviews of Diagnostic Test Accuracy 5 and reported according to the Preferred Reporting Items for Systematic Reviews and Meta-Analyses (PRISMA) guidelines 6 7 . The protocol and methods were discussed and finalized by the Committee on Confocal Laser Endomicroscopy of the Upper Gastrointestinal Tract prior to initiation of the review, considering the study objectives. The committee was composed of experienced pathologists, gastroenterologists, methodological experts, and biostatisticians to ensure multidisciplinary rigor and methodological validity. This review was not prospectively registered due to institutional constraints at the time of study initiation. However, all methods were defined a priori by a multidisciplinary expert panel and adhered to PRISMA-DTA guidelines. This review adheres to the PRISMA 2020 reporting guideline. A completed PRISMA checklist is provided in **Supplementary Table 1** .

### Search strategy

We performed a comprehensive literature search across Ovid-MEDLINE, Ovid-EMBASE, and the Cochrane Central Register of Controlled Trials from database inception to October 2024. The search strategy combined terms related to confocal microscopy and upper gastrointestinal neoplasia using Boolean operators, and truncation where appropriate. Representative keywords included: "Confocal endomicroscopy” AND (“Barrett esophagus” OR “Esophageal neoplasms” OR “Stomach Neoplasms” OR ((esophag* or gastr*) AND (lesion or dysplas*))). Controlled vocabulary (e.g., Medical Subject Headings [MeSH], Emtree) was applied as available. Search terms were finalized following a consensus review by the committee, and methods were appropriately applied and tailored to each database. In addition, domestic databases including KoreaMed, KMBASE, and RISS were searched using simplified Korean-language equivalents of the same conceptual terms. A manual search of reference lists from relevant systematic reviews and included articles was also conducted to identify additional eligible studies. Full electronic search strategies are detailed in **Supplementary Table 2** . No language restrictions were applied during the initial search phase, but inclusion was limited to studies published in English or Korean during the eligibility screening.

### Study selection and data extraction

All retrieved records were independently screened by two reviewers in a two-step process. First, titles and abstracts were evaluated for relevance. Full-text articles were then assessed against predefined eligibility criteria. Discrepancies were resolved by discussion, and unsolved conflicts were adjudicated by a third reviewer from the evaluation committee. Studies were included if they met all of the following criteria: 1) involved adult patients with suspected or confirmed esophageal or gastric neoplasia; 2) utilized pCLE as the index test; 3) used histopathology as the reference standard; and 4) reported diagnostic accuracy or relevant clinical outcomes. We excluded non-original articles, preclinical studies, conference abstracts, and duplicate reports using the same patient population. While we acknowledged the potential for heterogeneity stemming from diverse diagnostic thresholds (e.g., Miami classification), varying levels of operator expertise, and different interpretation workflows (real-time vs. offline), these factors were not used as exclusion criteria to maintain a broad and comprehensive clinical overview.

Data extraction was performed using a standardized and piloted form. One reviewer performed the initial abstraction, which was subsequently verified by a second reviewer. Extracted data included study characteristics (e.g., author, year, country, study design), patient demographics, details of the index and comparator tests, reference standards, and outcome measures. In cases of discrepancy between the enrolled and analyzed sample sizes, the analyzed population was used for synthesis. To address potential unit-of-analysis errors and within-patient clustering effects, we established a strict data extraction hierarchy. When included studies reported diagnostic accuracy across multiple analytical units (e.g., patient, lesion, or biopsy levels), per-patient data were preferentially extracted as the primary unit of analysis. Lesion- or biopsy-level data were extracted and utilized only when patient-level data were strictly unavailable.

### Endpoints and definitions

The primary outcome was diagnostic accuracy of pCLE, measured by sensitivity, specificity, and overall accuracy in detection of upper gastrointestinal dysplasia or malignancy. Secondary outcomes included additional lesion detection, impact on clinical management (such as changes in treatment plan or reduction in the number of biopsies), and safety endpoints, defined as incidence of procedure-related adverse events (AEs).

### Quality assessment

Methodological quality and risk of bias of included diagnostic accuracy studies were assessed using the Quality Assessment of Diagnostic Studies-2 (QUADAS-2) tool, in accordance with Cochrane Diagnostic Test Accuracy guidelines 8 . This tool evaluates risk of bias and concerns regarding applicability across four domains: patient selection, index test, reference standard, and flow and timing. Two independent reviewers conducted quality assessment of each study. Disagreements were resolved through discussion and, if needed, by consensus with a third reviewer from the study committee. Each domain was rated as having low, high, or unclear risk of bias and applicability concerns, using predefined signaling questions and domain-specific guidance. Particular attention was paid to: 1) blinding between the index test (pCLE) and the reference standard (histopathology); 2) patient spectrum and recruitment method (e.g., consecutive vs. selective inclusion); and 3) timing between the index test and reference standard, to ensure appropriate diagnostic comparison. A detailed visualization of the domain-level risk of bias and applicability concerns is presented in **Supplementary Fig. 1** .

### Statistical analysis

All statistical analyses were performed using R (version 4.2.2; R Foundation for Statistical Computing, Vienna, Austria) in RStudio (Posit Software, Boston, Massachusetts, United States) and Stata/MP 17.0 (StataCorp LLC, College Station, Texas, United States). Statistical significance was set at a two-sided *P* < 0.05.

To synthesize diagnostic performance of pCLE, we calculated pooled estimates of sensitivity and specificity using both the Mantel-Haenszel method and a bivariate random-effects meta-analytic model. The bivariate model jointly accounts for the correlation between sensitivity and specificity across studies and accommodates heterogeneity by modeling study-level random effects. This approach is recommended for diagnostic test accuracy reviews because of to its superior handling of threshold effects and study variability. Furthermore, to evaluate robustness of our pooled estimates and to mitigate risk of artificially inflated precision from correlated observations (clustering effect), we performed a pre-specified sensitivity analysis restricted exclusively to studies reporting patient-level data.

Forest plots were constructed to visually display individual study estimates and pooled values for sensitivity and specificity. Summary receiver operating characteristic (SROC) curves were generated, and the area under the curve (AUC) was calculated to quantify overall diagnostic accuracy.

Heterogeneity across studies was assessed using both the I ^2^ statistic and τ ^2^ . I ^2^ values > 50% were considered to suggest substantial heterogeneity, whereas τ ^2^ was reported as an absolute estimate of between-study variance. To evaluate potential publication bias, we applied Deeks’ funnel plot asymmetry test for diagnostic odds ratios (ORs). Subgroup analyses were performed based on lesion type and anatomical location (esophageal vs. gastric neoplasia) to explore sources of heterogeneity and assess consistency of pCLE performance across clinical scenarios.

Where applicable, comparative diagnostic accuracy between pCLE and conventional modalities (e.g., WLE, NBI) was summarized using paired accuracy metrics and visualized through head-to-head forest plots and SROC overlays.

## Results

### Search results and baseline characteristics

**Fig. 1:**
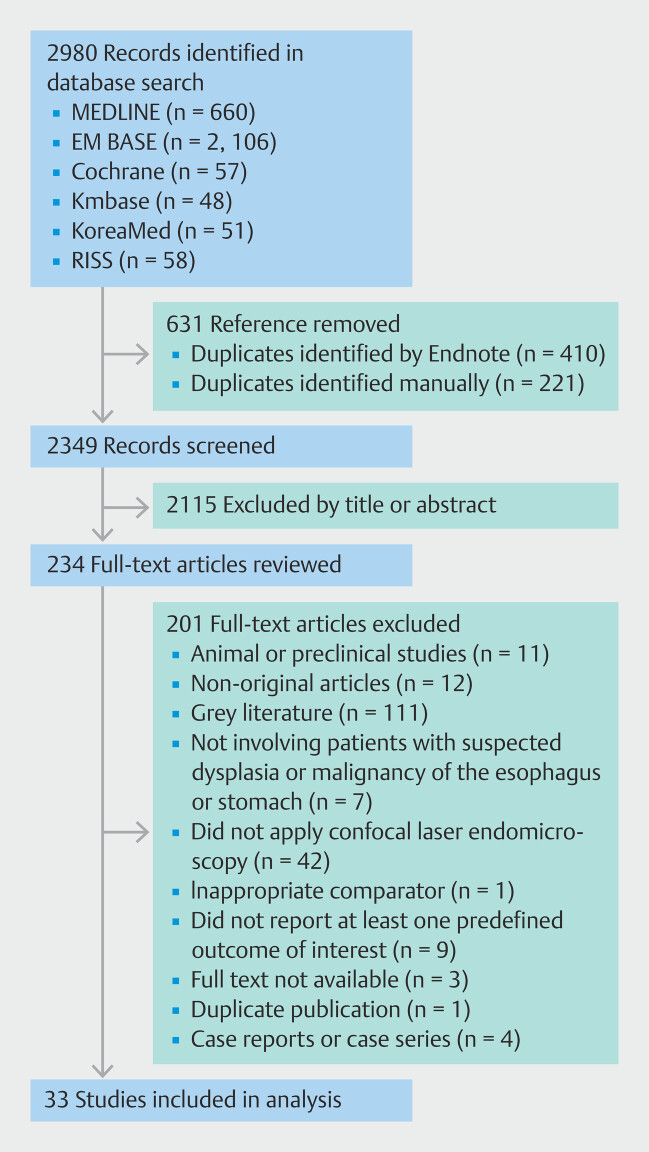
Preferred Reporting Items for Systematic Reviews and Meta-Analyses (PRISMA) flow diagram with systematic review of detailed searches 6 .

As shown in the PRISMA flow diagram ( Fig. 1 ), the initial search yielded 2,980 studies. After removal of duplicate records and exclusions based on title and abstract, 234 articles were fully reviewed. Of these, 201 citations were excluded because they did not meet inclusion criteria. After study selection, 33 studies were finally included: 30 assessed diagnostic accuracy and three were randomized clinical trials (RCTs) that did not report diagnostic metrics. Of the included studies, 15 focused on esophageal lesions, 16 on gastric lesions, and two on upper gastrointestinal lesions involving both sites. Sample sizes ranged from 14 to 356 patients, with 20 to 874 specimens. In most studies, pCLE was performed during WLE, with additional image-enhancement techniques such as NBI (10 studies), autofluorescence imaging (AFI, 2 studies), and flexible spectral imaging color enhancement (FICE, 2 studies) used in some cases. Comparator tests included other endoscopic modalities or pre-diagnostic biopsy results, whereas reference standards were based on histopathology from surgical or targeted biopsy specimens. Study characteristics are reported in Table 1 .

**Table:** **Table 1** Study design, clinical setting, and endoscopic modalities of included studies evaluating pCLE for upper gastrointestinal neoplasia.

First author, (year) [ref]	Country	Study design	Indications for CLE	Number of patients	Number of lesions	Modality	
						Index test	Comparator
Esophageal neoplasia							
Vithayathil (2022) 9	UK	RCT, crossover	Non-dysplastic Barrett's esophagus	134		HRWLE + AFI-guided pCLE	HRWLE + Seattle protocol
Krajciova (2020) 10	Czech Republic	Prospective	Post-endoscopic treatment surveillance (BERN)	56		HD-WLE + pCLE	Biopsies
Prueksapanich (2019) 11	Thailand	Cross-sectional	Surveillance after HNC	24	34	NBI + LCE + pCLE	WLE + dNBI
Richardson (2019) 12	USA	Prospective	BE screening/surveillance	172		WLE + NBI + pCLE	–
Shah (2018) 13	USA	Retrospective	Barrett’s esophagus	66		HD-WLE + NBI + pCLE	–
Caillol (2017) 14	France	Retrospective	Barrett’s esophagus	31		pCLE	Pre-resection biopsy (Seattle protocol)
Safatle-Ribeiro (2017) 15	Brazil	Prospective	Head and neck cancer	27		WLE + NBI + Chromoendoscopy with Lugol’s solution+pCLE	-
di Pietro (2015) 16	UK	Prospective	Barrett’s esophagus	55		AFI-targeted pCLE	AFI-targeted NBI (optical biopsy)
Guo (2015) 17	China	Prospective	Early esophageal neoplasia	356		WLE + pCLE	I-Scan alone, I-Scan + pCLE
Prueksapanich (2015) 18	Thailand	Cross-sectional, crossover	Head and neck cancer history, no dysphagia	44		WLE + pCLE	dNBI
Bertani (2013) 19	Italy	Prospective	Barrett’s esophagus	100 (index test 50, comparator 50)		HD-WLE + pCLE	HD-WLE
Wallace (2012) 20	USA	RCT	Barrett’s esophagus	164 (HDWL + pCLE 82; HDWL only 82)		HDWL + pCLE	HDWL
Sharma (2011) 21	USA	RCT	Barrett’s esophagus	101		pCLE alone, HD-WLE + pCLE, HD-WLE + NBI + pCLE	HD-WLE alone, NBI alone, HD-WLE + NBI
Bajbouj (2010) 22	Germany	Prospective	Barrett’s esophagus	68		HD-WLE + pCLE	–
Pohl (2008) 23	Germany	Prospective	Barrett’s esophagus	38	296	High-resolution videoendoscopy + Miniprobe confocal laser microscopy	–
Gastric neoplasia							
Pilonis (2023) 24	UK	Prospective	Hereditary diffuse gastric cancer	31		WLI + NBI + pCLE	–
Kim (2022) 25	South Korea	Prospective	Gastric cancer under chemotherapy	23		WLE/M-NBI + pCLE	WLE/M-NBI
Pang (2022) 26	China	Prospective	Atrophic gastritis	21		WLE + pCLE	–
Chu (2021) 27	China	Retrospective	Early gastric cancer and precancerous lesions	226 (sedated 126, unsedated 100)		WLE + pCLE	–
Schueler (2021) 28	USA	Prospective	Hereditary diffuse gastric cancer	36		HD-WLE + pCLE	Cambridge method (non-targeted gastric biopsies)
Park d(2019) 29	South Korea	RCT	Biopsy-proven poorly cohesive carcinoma	Pilot (n=30): pCLE 15, WLE 15 Confirmatory (n=61): pCLE 29, WLE 32		WLE + pCLE	WLE-based biopsy
Chen (2018) 30	China	Retrospective	Suspected upper gastrointestinal tract lesions	322		WLE + pCLE	–
Horiguchi (2018) 31	Japan	Prospective	Early gastric cancer	30	36	WLE + ME-NBI + pCLE	WLE, ME-NBI
Park (2017) 32	South Korea	RCT	Differentiated EGC or dysplasia	78 (index test 36, comparator 42)		pCLE	WLI with chromoendoscopy
Zuo (2017) 33	China	RCT	Post-Hp gastritis or GIM/GIN	238 (FICE + pCLE: 120, FICE: 118)		FICE-guided pCLE	FICE with standard biopsies
Kobayashi (2016) 34	Japan	Prospective	Gastric lesions	30	45	WLE + pCLE	WLE
Li (2016) 35	China	Prospective	Dyspepsia, Hp infection, or gastric neoplasia	240		HDE + pCLE	–
Gong (2015) 36	China	Prospective	Suspected gastric cancer	82	86	WLE + pCLE	WLE + ME-NBI
Bok (2013) 37	South Korea	Prospective	Gastric neoplasia	46	54	HDE + pCLE	Forceps biopsy
Lim (2013) 38	Singapore	RCT	Past history of GIM	20		WLE + AFI/mNBI + pCLE	WLE, AFI, mNBI
Pittayanon (2013) 39	Thailand	Prospective	Confirmed GIM	60		ME-FICE + pCLE	–
Upper GI (mixed)							
Seerani (2023) 40	Pakistan	Prospective	uncertain upper GI diagnosis	90		WLE + pCLE	–
Kollar (2020) 41	Czech Republic	Prospective	Esophageal or gastric lesions	67	74	HD-WLE + NBI + pCLE	Forceps biopsies
BERN, Barrett’s-related esophageal neoplasia; dNBI, dual-focus narrow-band imaging; FICE, flexible imaging color enhancement; GIM, gastric intestinal metaplasia; GIN, gastric intraepithelial neoplasia; HDGC, hereditary diffuse gastric cancer; HDWL, high-definition white-light endoscopy; Hp, Helicobacter pylori; I-Scan, high-definition virtual chromoendoscopy; IQR, interquartile range; ME-NBI, magnifying endoscopy with narrow-band imaging; RCT, randomized controlled trial; WLE, white-light endoscopy; WLI, white-light imaging.							

### Diagnostic accuracy

Because the included studies variably reported diagnostic accuracy at the patient, lesion, biopsy, or specimen level, the revised synthesis prioritized patient-level data whenever available and supplemented the primary analyses with prespecified sensitivity analyses. In the broader organ-based analyses that incorporated all eligible revised datasets, pooled sensitivity and specificity were 0.89 (95% confidence interval [CI] 0.84–0.92) and 0.79 (95% CI 0.68–0.87), respectively, for esophageal lesions, and 0.89 (95% CI 0.83–0.93) and 0.95 (95% CI 0.92–0.96), respectively, for gastric lesions ( **Supplementary Fig. 2** and **Supplementary Fig. 3** ). Corresponding AUC values were 0.897 for the esophageal analysis and 0.960 for the gastric analysis. Diagnostic accuracy of pCLE across different conditions and comparative modalities is summarized in Table 2 .

**Table:** **Table 2** Summary of diagnostic accuracy of pCLE in broader organ-based analyses.

Condition	Sensitivity (pCLE)	Specificity (pCLE)	Comparator (WLE/NBI)	Notes
Esophageal lesions	0.89 (95% CI 0.84–0.92)	0.79 (95% CI 0.68–0.87)	Higher sensitivity in direct comparisons	Broader organ-based analysis including all eligible esophageal-target studies; see **Supplementary Fig. 2**
Gastric lesions	0.89 (95% CI 0.83–0.93)	0.95 (95% CI 0.92–0.96)	Comparable or modestly improved specificity in direct comparisons	Broader organ-based analysis including all eligible gastric-target studies; see **Supplementary Fig. 3**
CI, confidence interval; NBI, narrow-band imaging; pCLE, probe-based confocal laser endomicroscopy; WLE, white-light endoscopy.				

**Fig. 2:**
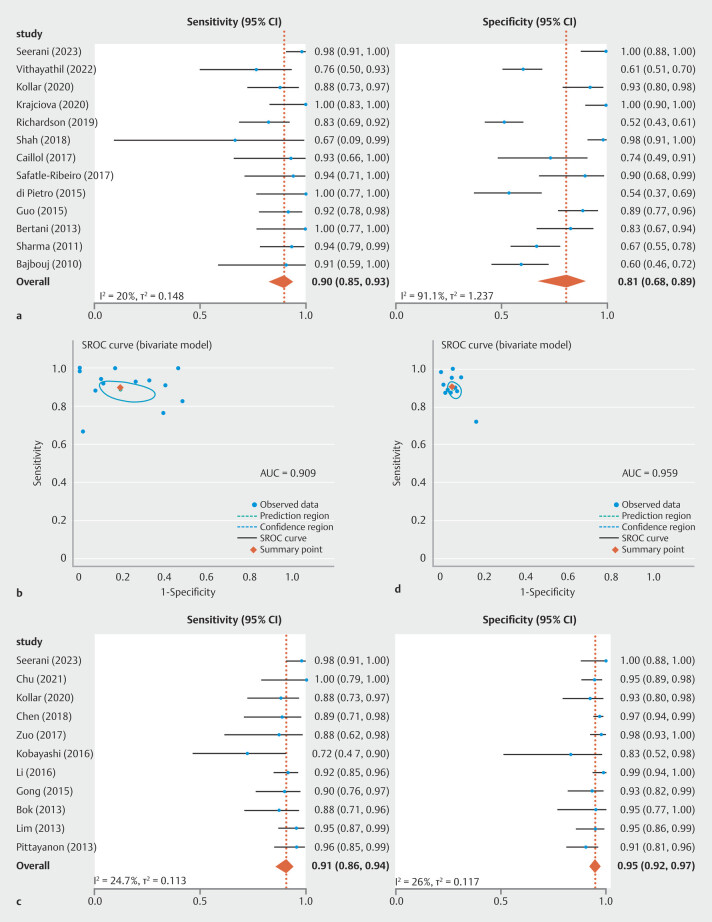
Diagnostic accuracy of probe-based confocal laser endomicroscopy for upper gastrointestinal neoplasia by anatomic site. **a** Forest plot of pooled sensitivity and specificity for esophageal neoplasia. **b** Summary receiver operating characteristic (SROC) curve for esophageal neoplasia. **c** Forest plot of pooled sensitivity and specificity for gastric neoplasia. **d** SROC curve for gastric neoplasia. Pooled estimates were derived from bivariate random-effects models. Between-study heterogeneity is described using both I2 and τ2. Broader organ-based analyses and per-patient sensitivity analyses are shown in **Supplementary Fig.4** and **Supplementary Fig.5** . pCLE, probe-based confocal endomicroscopy; WLE, white-light endoscopy.

Because Deeks’ test suggested small-study effects in gastric analyses, additional sensitivity analyses excluding studies with fewer than 30 patients were performed. These analyses yielded materially similar results. For esophageal neoplasia, pooled sensitivity was 0.90 (95% CI 0.85–0.93) and pooled specificity was 0.81 (95% CI 0.68–0.89), with an AUC 0.909 ( Fig. 2 **a** and Fig. 2 **b** ). For gastric neoplasia, pooled sensitivity was 0.91 (95% CI 0.86–0.94) and pooled specificity was 0.95 (95% CI 0.92–0.97), with an AUC of 0.959 ( Fig. 2 **c** and Fig. 2 **d** ). These findings indicate that exclusion of smaller studies did not materially alter the overall estimates.

In sensitivity analyses restricted to studies reporting per-patient data, the pooled estimates remained directionally consistent, although CIs widened, as expected. In esophageal studies, pooled sensitivity and specificity were 0.89 (95% CI 0.81–0.94) and 0.76 (95% CI 0.58–0.88), respectively, with an AUC of 0.905 ( **Supplementary Fig. 4)** . In gastric studies, pooled sensitivity and specificity were 0.85 (95% CI 0.68–0.94) and 0.94 (95% CI 0.88–0.97), respectively, with an AUC of 0.944 ( **Supplementary Fig. 5** ). Taken together, these findings suggest that the overall diagnostic conclusions were robust to the analytic unit used, although mixed-unit pooling may overstate precision.

Head-to-head comparisons between pCLE and conventional endoscopic modalities are summarized in **Supplementary Table 3** . Across the three studies directly comparing pCLE with WLE, pCLE consistently demonstrated higher sensitivity (range 0.68–0.91), with comparable or modestly improved specificity (range 0.83–0.88). These comparative data support the role of pCLE as an adjunctive characterization tool rather than a replacement for high-quality conventional endoscopy.

### Subgroup analysis by lesion type

**Fig. 3:**
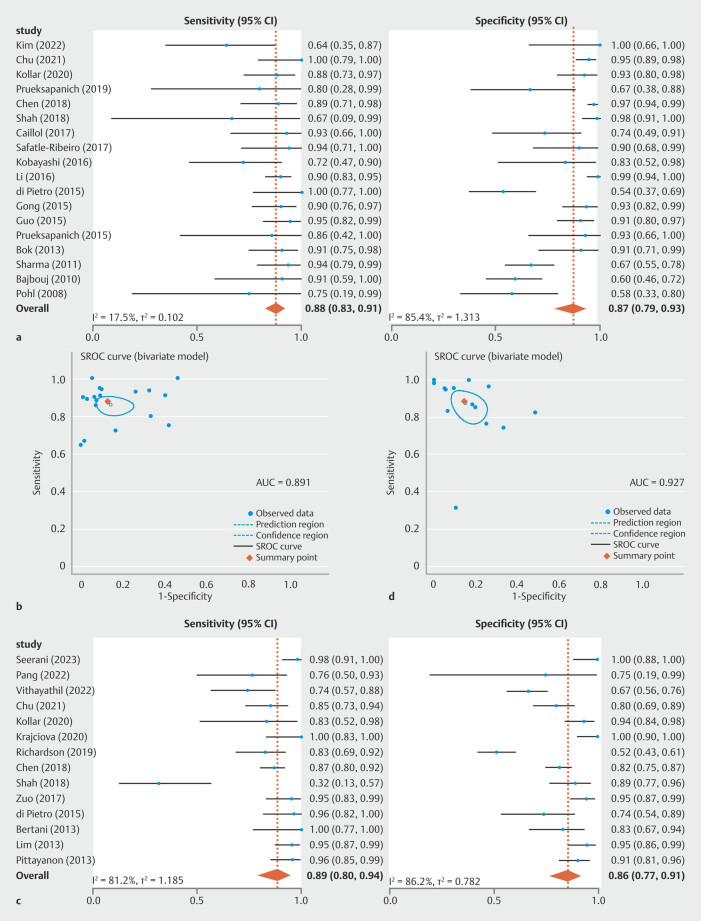
Diagnostic accuracy of probe-based confocal laser endomicroscopy according to lesion category. **a** Forest plot of pooled sensitivity and specificity for high-grade or frankly neoplastic lesions, including high-grade dysplasia, early carcinoma, and invasive adenocarcinoma. **b** SROC curve for high-grade or frankly neoplastic lesions. **c** Forest plot of pooled sensitivity and specificity for lower-grade or metaplastic lesions, including low-grade dysplasia and gastric intestinal metaplasia. **d** SROC curve for lower-grade or metaplastic lesions. Pooled estimates were derived from bivariate random-effects models. Lower-grade/metaplastic lesions showed substantially greater between-study heterogeneity and wider dispersion despite preserved overall discrimination. Per-patient sensitivity analyses are presented in **Supplementary Fig. 6** and **Supplementary Fig. 7** .

Diagnostic accuracy of pCLE varied meaningfully by lesion type ( Table 3 , Fig. 3 ). For high-grade or frankly neoplastic lesions, including HGD, early carcinoma, and invasive adenocarcinoma, pooled sensitivity was 0.88 (95% CI 0.83–0.91) and pooled specificity was 0.87 (95% CI 0.79–0.93) ( Fig. 3 **a** ). The corresponding SROC curve yielded an AUC of 0.89, indicating strong overall discrimination for advanced or overtly neoplastic targets ( Fig. 3 **b** ). Heterogeneity was modest for sensitivity but substantial for specificity, indicating that although pCLE was generally effective in advanced or overtly neoplastic lesions, performance still varied across clinical settings and interpretation frameworks.

**Table:** **Table 3** Diagnostic accuracy of pCLE by lesion type.

Author (Year)	Modality	Lesion	True positive	False positive	False negative	True negative	Sensitivity	Specificity
Seerani (2023)	WLE + pCLE	Upper gastrointestinal lesions	59	0	1	30	0.98	1.00
Pang (2022)	WLE + pCLE	GIM	13	1	4	3	0.76	0.75
Vithayathil (2022)	AFI guided pCLE	Dysplasia	26	33	9	66	0.74	0.67
Chu (2021)	WLE + pCLE	GIM	47	14	8	57	0.85	0.80
Kollar (2020)	HD-WLE + NBI + pCLE	Dysplasia	10	4	2	58	0.83	0.94
Krajciova (2020)	HD-WLE + pCLE	GIM	20	0	0	36	1.00	1.00
Richardson (2019)	WLE + NBI + pCLE	GIM	38	61	8	65	0.83	0.52
Chen (2018)	WLE + pCLE	Atrophy and/or GIM	132	31	20	139	0.87	0.82
Shah (2018)	HD-WLE + NBI + pCLE	LGD	6	5	13	42	0.32	0.89
Zuo (2017)	FICE-guided pCLE	GIM	38	4	2	70	0.95	0.95
di Pietro (2015)	AFI + pCLE	LGD + HGD/IMC	27	7	1	20	0.96	0.74
Bertani (2013)	HD-WLE + pCLE	HGD and LGD	14	6	0	30	1.00	0.83
Lim (2013)	pCLE	GIM	60	9	6	50	0.91	0.85
Pittayanon (2013)	ME-FICE + pCLE	GIM	44	7	2	67	0.96	0.91
Kim (2022)	WLE/M-NBI + pCLE	Residual cancer tissues	9	0	5	9	0.64	1.00
Chu (2021)	WLE + pCLE	EGC	16	6	0	104	1.00	0.95
Schueler (2021)	HD-WLE + pCLE	SRC	5	0	10	0	0.33	
Kollar (2020)	HD-WLE + NBI + pCLE	Malignant lesions	30	3	4	37	0.88	0.93
Prueksapanich (2019)	NBI + LCE + pCLE	ESCN	4	5	1	10	0.80	0.67
Chen (2018)	WLE + pCLE	Adenocarcinoma	24	9	3	286	0.89	0.97
Shah (2018)	HD-WLE + NBI + pCLE	HGD or cancer	2	1	1	62	0.67	0.98
Caillol (2017)	pCLE	HGD/EAC	13	5	1	14	0.93	0.74
Safatle-Ribeiro (2017)	WLE + NBI + Chromoendoscopy with Lugol solution + pCLE	ESCN	16	2	1	18	0.94	0.90
Kobayashi (2016)	WLE + pCLE	Neoplastic or non-neoplastic	13	2	5	10	0.72	0.83
Li (2016)	HD-WLE + pCLE	IEN + IMC	98	1	11	88	0.90	0.99
di Pietro (2015)	AFI + pCLE	HGD/IMC	194	64	0	130	1.00	0.67
Gong (2015)	WLE + pCLE	Cancerous/noncancerous	36	3	4	43	0.90	0.93
Guo (2015)	WLE + pCLE	Esophageal SCC or IEN	35	5	2	49	0.95	0.91
Prueksapanich (2015)	WLE + pCLE	Early ESCN	6	1	1	13	0.86	0.93
Bok (2013)	HD-WLE + pCLE	Adenocarcinoma (vs. non-neoplastic or dysplasia)	29	2	3	20	0.91	0.91
Sharma (2011)	HD-WLE + pCLE	HGD/EC	82	92	38	662	0.68	0.88
Bajbouj (2010)	HD-WLE + pCLE	HGD/carcinoma	16	19	42	626	0.28	0.97
Pohl (2008)	HD-WLE + pCLE	HGD /EC	10	10	2	157	0.83	0.94
AFI, autofluorescence imaging; EAC, esophageal adenocarcinoma; EC, early carcinoma; EGC, early gastric cancer; ESCN, esophageal squamous cell neoplasia; FICE, flexible spectral imaging color enhancement; FN, false negative; FP, false positive; GIM, gastric intestinal metaplasia; HGD, high-grade dysplasia; HD-WLE, high-definition white-light endoscopy; IEN, intraepithelial neoplasia; IM, intestinal metaplasia; IMC, intramucosal carcinoma; LGD, low-grade dysplasia; ME, magnifying endoscopy; ME-FICE, magnifying flexible spectral imaging color enhancement; NBI, narrow-band imaging; pCLE, probe-based confocal laser endomicroscopy; SCC, squamous cell carcinoma; SRC, signet-ring cell carcinoma; WLE, white-light endoscopy.								

For lower-grade or metaplastic lesions, including low-grade dysplasia (LGD) and GIM, pooled sensitivity was 0.89 (95% CI 0.80–0.94) and pooled specificity was 0.86 (95% CI 0.77–0.91) ( Fig. 3 **c** ), with an AUC of 0.927 on SROC analysis ( Fig. 3 **d** ). However, this subgroup showed substantially greater between-study heterogeneity and broader dispersion, suggesting that pCLE performance in subtle lesions was more dependent on lesion phenotype, diagnostic thresholds, and clinical context.

Per-patient restricted subgroup sensitivity analyses were directionally consistent but slightly attenuated. For high-grade or frankly neoplastic lesions, pooled sensitivity and specificity were 0.84 (95% CI 0.73–0.91) and 0.83 (95% CI 0.66–0.92), respectively, with an AUC of 0.865 ( **Supplementary Fig. 6** ). For lower-grade or metaplastic lesions, pooled sensitivity and specificity were 0.86 (95% CI 0.72–0.93) and 0.79 (95% CI 0.69–0.87), respectively, with an AUC of 0.880 ( **Supplementary Fig. 7** ). These findings further support the interpretation that pCLE performs best in overt neoplasia and that estimates in subtle lesions should be interpreted more cautiously.

### Additional lesion detection

Eight studies investigated incremental diagnostic yield of pCLE to identify neoplastic foci missed by conventional imaging ( **Supplementary Table 4** ). In the context of BE surveillance, adjunctive use of pCLE with high-definition WLE (HD-WLE) significantly increased the per-patient detection rate of dysplasia from 10% (5/50) to 28% (14/50) ( *P* = 0.04) ( **Supplementary Fig. 8** ). Similarly, another multicenter trial reported a 133% increase in detection of neoplasia compared with HD-WLE alone (7/62 vs. 3/57 patients).

In gastric cancer settings, specifically for patients receiving chemotherapy, pCLE-targeted biopsies achieved a significantly higher inclusion rate of malignant tissue per biopsy (74.5%, 41/55) compared with WLE/M-NBI (52.2%, 32/55). Furthermore, for detecting poorly cohesive adenocarcinoma, pCLE-guided biopsies demonstrated a superior detection rate (65%, 19/29) over standard WLE-directed biopsies (30%, 10/32) ( *P* = 0.01). In addition, in patients with suspected gastric precancerous lesions, a randomized trial demonstrated a significantly higher diagnostic yield per biopsy when pCLE was combined with FICE compared with FICE alone (75.1%, 313/417 vs. 31.5%, 252/800; *P* <0.001).

### Impact on clinical management

Impact of pCLE on clinical decision-making was synthesized narratively because definitions of management change, baseline comparators, and denominators differed substantially across studies. To improve transparency, study-level details regarding setting, comparator modality, denominator, unit of analysis, and direction of management effect are summarized in **Supplementary Table 5** . In a highly selected cohort of 13 patients with BE and subtle mucosal irregularities without a discrete macroscopic lesion, pCLE altered real-time therapeutic management in 69.2% (9/13) by either prompting endoscopic resection for suspected higher-grade disease or avoiding unnecessary endoscopic resection or radiofrequency ablation when neoplasia was not supported. In addition, three studies reported that pCLE-guided targeting reduced biopsy burden. In one randomized study of patients with suspected gastric precancerous lesions, FICE-guided pCLE reduced the mean number of biopsies per patient by 48.5% compared with FICE alone (3.5 vs. 6.8; *P* <0.001). In another study of esophageal squamous neoplasia, the high negative predictive value of pCLE allowed biopsy to be avoided in 51 of 91 suspected lesions (56.0%). These findings support the potential adjunctive clinical utility of pCLE, but they should be interpreted as setting-specific rather than as standardized pooled estimates of management effect.

### Safety profile

Of the 33 included studies, 11 explicitly reported no AEs. Mild complications included minor bleeding in nine of 209 patients (4.3%) across four studies and contrast-related allergic reactions in one of 56 patients (1.8%) across two studies. These events were managed conservatively with standard supportive treatment. One study reported serious events including perforation and aspiration pneumonia, in a patient who underwent both pCLE and chromoendoscopy, but no difference in complication rates was observed between the two modalities. These findings suggest that pCLE has an acceptable safety profile, comparable to standard endoscopic procedures.

### Quality assessment

Methodological quality was assessed using QUADAS-2 and the results are summarized in **Supplementary Fig. 1** . Overall, most studies were judged to have a low risk of bias in patient selection, reference standard, and flow/timing domains. However, several studies had high or unclear risk of bias in the index-test domain, primarily due to incomplete reporting of blinding, threshold definition, or interpretation workflow. Applicability concerns were generally low across all studies.

### Assessment of publication bias

Publication bias was assessed using Deeks’ funnel plot asymmetry test based on diagnostic ORs. Deeks’s test did not provide strong statistical evidence of funnel plot asymmetry ( *P* = 0.12) for esophageal studies. However, given the limited number of studies and the known low power of asymmetry tests in small meta-analyses, publication bias/small-study effects cannot be excluded ( **Supplementary Fig. 9a** ).

Conversely, in studies focusing on gastric dysplasia or malignancy, the funnel plot showed marked asymmetry and the Deeks’ test yielded a statistically significant result ( *P* = 0.01), suggesting potential small-study effects and publication bias ( **Supplementary Fig. 9b** ). These findings indicate that pooled estimates for gastric lesions should be interpreted with caution because studies reporting higher diagnostic accuracy may be overrepresented.

### Discussion

In this systematic review of 33 studies (30 diagnostic accuracy and three RCTs), pCLE demonstrated consistently high diagnostic accuracy for upper gastrointestinal neoplasia. When referenced to histopathology, pooled sensitivity and specificity were 0.89 for esophageal lesions and ≥ 0.90 for gastric lesions, placing pCLE on par with or slightly superior to, high-resolution imaging such as WLE and NBI. Beyond its baseline performance, pCLE demonstrated additive value by uncovering clinically relevant neoplastic foci that were missed by conventional endoscopy. This capability enhances lesion targeting and may lead to improved detection of histologically aggressive cancers, ultimately supporting more tailored therapeutic strategies. These findings highlight the “add-on” diagnostic value of pCLE, particularly in settings where conventional imaging yields equivocal results. Its safety profile was comparable to standard endoscopy, with only minor and manageable AEs reported. This suggests that pCLE may offer diagnostic enhancement without compromising procedure safety, which is particularly relevant for patients requiring repeated surveillance or who are at higher risk for biopsy-related complications.

Our pooled estimates are slightly higher than earlier analyses that reported sensitivities of 72% to 91% and specificities of 84% to 99% 42 43 . The upward trend likely reflects multiple converging factors such as implementation of second-generation probes with enhanced resolution and optical penetration, increased operator familiarity and structured training programs, and broader adoption of validated interpretation systems for microarchitectural patterns such as the Miami and Paris classifications 44 45 . These technological and procedural refinements have enhanced lesion detectability and interobserver reliability.

Importantly, matched comparisons across studies consistently showed that pCLE outperformed WLE in sensitivity, with relative gains reaching up to 53% in some datasets. Specificity was similar or modestly improved, suggesting a lower false-positive rate. pCLE’s ability to visualize subepithelial structures and microvasculature in real time provides key advantages over surface-based imaging, especially for early neoplastic changes 1 . Real-time assessment of mucosal architecture enables dynamic adjustment of the endoscopic view. This facilitates more precise targeting and reduces reliance on random biopsies. Indeed, in up to 70% of BE cases, pCLE reclassified dysplasia grade, prompting management changes. This real-time histologic insight allows clinicians to adjust treatment strategy on the spot, either avoiding unnecessary resection or escalating therapy appropriately, leading to more efficient and patient-centered decision-making.

Subgroup analyses indicated that pCLE diagnostic performance varies by lesion type. Although its accuracy is excellent for HGD or overt neoplasia, performance for GIM and LGD was more modest. Interpretation heterogeneity is a key limitation: across included studies, pCLE positivity thresholds and diagnostic criteria were variably defined (e.g., Miami classification versus study-specific definitions or incompletely reported rules) 44 , and diagnostic performance, therefore, is better understood as context-dependent rather than device-intrinsic. In addition, because pCLE is reader-dependent and workflows differ (real-time bedside interpretation vs offline review), variability in reader expertise/training and reading conditions likely contributed to between-study heterogeneity and may limit generalizability of pooled estimates beyond expert-center settings 46 . From a clinical standpoint, understanding variable diagnostic performance across lesion types helps set realistic expectations and guides appropriate indications for pCLE use in practice. Artificial intelligence (AI)-assisted classification tools, trained on large annotated datasets, may help enhance diagnostic confidence in such settings 47 .

Fig. 3**a** and Fig. 3 **c** present pooled sensitivity and specificity estimates for high-grade/frankly neoplastic lesions and lower-grade/metaplastic lesions, respectively, whereas Fig. 3 **b** and Fig. 3 **d** show the corresponding SROC curves. Although neoplastic lesions cluster tightly in the high-accuracy quadrant, lower-grade lesions such as GIM and LGD show greater dispersion, reflecting variability in diagnostic performance. Such lesion-dependent accuracy trends call for structured diagnostic pathways and signal an opportunity for technology-driven support systems to assist less experienced endoscopists.

The review's strength is its consistent accuracy estimates across diverse organ sites, despite geographical and methodological variability. Limitations include 16% to 20% of studies exhibiting unclear or high bias risks in the “index test” and “reference standard” domains per QUADAS-2. In addition, although we prioritized patient-level data extraction, some included studies only provided lesion-level data. The potential for uncontrolled intra-patient clustering effects in these specific studies remains a limitation because it could marginally inflate the precision of our pooled estimates.

Publication bias and small-study effects represent a meaningful limitation of this meta-analysis. For esophageal studies, Deeks’ test did not provide strong statistical evidence of funnel plot asymmetry. However, given the low power of asymmetry tests in small meta-analyses, a statistically non-significant result should not be interpreted as proof of no bias 48 . Conversely, for gastric analyses, the significant Deeks’ test and asymmetric funnel plot suggest that small-study effects are present, implying overrepresentation of studies reporting higher pCLE diagnostic accuracy. Although our sensitivity analysis excluding smaller studies yielded highly robust estimates, maintaining a pooled gastric specificity of 0.95, it does not completely eliminate the possibility of reporting bias. For this reason, the gastric results should be interpreted as encouraging but not definitive. Claims based on exceptionally high specificity must be explicitly tempered and clinicians should cautiously interpret these pooled estimates, especially when applying them to broader patient criteria or less experienced operator settings.

To counteract publication bias in future analyses, several strategies are recommended. First, conducting sensitivity analyses that omit smaller studies can evaluate robustness of pooled accuracy estimates. Significant changes in results would indicate small-study bias effects. Second, prospective registration of diagnostic accuracy studies in public registries is crucial for enhancing transparency and reducing selective reporting. Third, incorporating gray literature may reveal underreported findings, albeit with careful methodological scrutiny. Lastly, advanced statistical techniques such as trim-and-fill may provide additional insights, although they have limitations requiring cautious interpretation.

Existing evidence primarily emerges from high-volume academic centers in Asia and Europe, potentially constraining generalizability to lower-prevalence or community-based settings. Future research should focus on prospective studies in varied clinical environments, including community hospitals and screening populations, to ascertain replicability of pCLE diagnostic accuracy in routine practice. Moreover, multicenter pragmatic trials comparing pCLE with existing imaging modalities, such as NBI, should be undertaken, evaluating diagnostic yield and patient-centered outcomes such as procedure burden, quality-adjusted life-years, and cancer-free survival. Beyond study design, implementation challenges must be addressed. The shortage of trained endoscopists is a significant barrier to widespread pCLE adoption. Establishing standardized training programs for pCLE image acquisition and interpretation, alongside certification processes, could enhance its accessibility. Furthermore, leveraging AI-assisted platforms for image interpretation may help standardize pCLE application by minimizing interobserver variability and aiding less experienced clinicians. These strategies could promote broader and more consistent application of pCLE across diverse healthcare settings.

Despite these limitations, pCLE offers unique value in specific clinical scenarios. These include targeted evaluation of inconspicuous or multifocal lesions missed by WLE or NBI, intra-procedural assessment of lateral margins in endoscopic resection, and surveillance in hereditary cancer syndromes or postsurgical settings where minimizing biopsy burden is critical. However, as recently synthesized by Ayyad et al 49 . widespread implementation is hindered by real-world adoption barriers, including high equipment costs, the need for specialized operator training, and extended procedure time. This highlights a crucial distinction between the high theoretical diagnostic potential of pCLE and its practical utility in routine clinical settings. Nevertheless, the potential to reduce unnecessary biopsies and improve diagnostic yield may offset these challenges by streamlining workflow and lowering cumulative procedural burden on patients and providers alike.

Our pooled estimates are limited by the fact that most studies were conducted in high-volume academic centers in Asia and Europe, where patient mix, disease prevalence, and operator expertise differ from routine practice. Because pCLE is reader-dependent, outcomes hinge on training, credentialing, workflow integration, and quality assurance. Broader adoption is often constrained by the learning curve, added procedure time, contrast use, and equipment costs, which pose greater challenges in lower-volume or community settings. Variability in interpretation frameworks (e.g., Miami vs. study-specific criteria) further reduces generalizability. Thus, our findings mainly reflect optimized expert-center conditions, and pragmatic multicenter studies are needed to assess real-world effectiveness, training needs, and economic impact.

Future research should prioritize multicenter pragmatic trials to compare pCLE and NBI strategies regarding diagnostic yields and clinical outcomes. Recent advancements in endoscopic imaging, such as BLI, linked color imaging (LCI), and AI-based systems, improve mucosal contrast and lesion detection. Nonetheless, these techniques do not achieve the subcellular resolution that pCLE offers. pCLE enables in vivo microscopic assessment, serving as an “optical biopsy” to guide clinical decisions in complex cases. Unlike BLI and LCI, which focus on enhancing macroscopic imaging, pCLE excels in lesion characterization and histological analysis. AI diagnostic tools, which are still in development, may complement pCLE by providing standardized image evaluations. Integration of AI with pCLE could address operator-dependent interpretation challenges, particularly in the context of early-stage pathology. Collectively, these imaging platforms address distinct diagnostic needs. BLI and LCI contribute to detection, pCLE supports characterization, and AI enables standardization. Integrating these tools in a tiered algorithm may yield optimal diagnostic efficiency.

In addition, prospective studies in low-prevalence populations are warranted to clarify pCLE’s negative predictive value and optimize patient selection criteria. Integrating lesion-specific performance characteristics, particularly the lower sensitivity for GIM and LGD, into clinical algorithms may enhance its judicious application. Refined diagnostic criteria, potentially informed by machine-learning models trained on large-scale image databases, could further enhance precision and clinical applicability.

## Conclusions

In conclusion, this review affirms that pCLE provides high diagnostic accuracy for upper gastrointestinal neoplasia and confers additional clinical benefits such as reduced biopsy burden and improved lesion detection. Although its safety is comparable to conventional endoscopy, its greatest utility lies in well-defined clinical contexts, namely, when standard imaging is inconclusive, biopsy sampling is constrained, or lesion-level characterization is needed. Addressing current limitations, such as cost, training, and workflow integration, and advancing AI-driven interpretation tools will be key to transitioning pCLE from a specialist adjunct to a standard component of modern endoscopic practice. Taken together, pCLE not only advances diagnostic capability but also enhances patient experience and care efficiency, making it a compelling candidate for broader integration into patient-centered endoscopy.

## Supplementary Material

Supplementary Material Zusatzmaterial
