# Supplementary material for: Confocal laser endomicroscopy for upper gastrointestinal neoplasia: Systematic review and meta-analysis
Source: Endosc Int Open. 2026 May 12;14:a28631407. doi: 10.1055/a-2863-1407 (PMC13289976; doi:10.1055/a-2863-1407)

Supplementary Materials
Supplement to: Diagnostic accuracy and clinical impact of confocal laser endomicroscopy in upper gastrointestinal neoplasia: Systematic review and meta-analysis
This document includes Supplementary Tables 1-5 and Supplementary Fig. 1-9.

Supplementary Tables
Supplementary Table 1 PRISMA 2020 checklist.

Section and Topic	Item #	Checklist item	Location where item is reported
**TITLE**			
Title	1	Identify the report as a systematic review.	p.1
**ABSTRACT**			
Abstract	2	See the PRISMA 2020 for Abstracts checklist.	See table below
**INTRODUCTION**			
Rationale	3	Describe the rationale for the review in the context of existing knowledge.	p.4
Objectives	4	Provide an explicit statement of the objective(s) or question(s) the review addresses.	p.4
**METHODS**			
Eligibility criteria	5	Specify the inclusion and exclusion criteria for the review and how studies were grouped for the syntheses.	p.5,6
Information sources	6	Specify all databases, registers, websites, organisations, reference lists and other sources searched or consulted to identify studies. Specify the date when each source was last searched or consulted.	p.5
Search strategy	7	Present the full search strategies for all databases, registers and websites, including any filters and limits used.	Supplementary Table 2
Selection process	8	Specify the methods used to decide whether a study met the inclusion criteria of the review, including how many reviewers screened each record and each report retrieved, whether they worked independently, and if applicable, details of automation tools used in the process.	p.5,6
Data collection process	9	Specify the methods used to collect data from reports, including how many reviewers collected data from each report, whether they worked independently, any processes for obtaining or confirming data from study investigators, and if applicable, details of automation tools used in the process.	p.6
Data items	10a	List and define all outcomes for which data were sought. Specify whether all results that were compatible with each outcome domain in each study were sought (e.g. for all measures, time points, analyses), and if not, the methods used to decide which results to collect.	p.6
	10b	List and define all other variables for which data were sought (e.g. participant and intervention characteristics, funding sources). Describe any assumptions made about any missing or unclear information.	p.6
Study risk of bias assessment	11	Specify the methods used to assess risk of bias in the included studies, including details of the tool(s) used, how many reviewers assessed each study and whether they worked independently, and if applicable, details of automation tools used in the process.	p.6
Effect measures	12	Specify for each outcome the effect measure(s) (e.g. risk ratio, mean difference) used in the synthesis or presentation of results.	p.7
Synthesis methods	13a	Describe the processes used to decide which studies were eligible for each synthesis (e.g. tabulating the study intervention characteristics and comparing against the planned groups for each synthesis (item #5)).	p.7
	13b	Describe any methods required to prepare the data for presentation or synthesis, such as handling of missing summary statistics, or data conversions.	p.7
	13c	Describe any methods used to tabulate or visually display results of individual studies and syntheses.	p.7
	13d	Describe any methods used to synthesize results and provide a rationale for the choice(s). If meta-analysis was performed, describe the model(s), method(s) to identify the presence and extent of statistical heterogeneity, and software package(s) used.	p.7
	13e	Describe any methods used to explore possible causes of heterogeneity among study results (e.g. subgroup analysis, meta-regression).	p.7

Section and Topic	Item #	Checklist item	Location where item is reported
	13f	Describe any sensitivity analyses conducted to assess robustness of the synthesized results.	p.7
Reporting bias assessment	14	Describe any methods used to assess risk of bias due to missing results in a synthesis (arising from reporting biases).	p.6
Certainty assessment	15	Describe any methods used to assess certainty (or confidence) in the body of evidence for an outcome.	p.7
**RESULTS**			
Study selection	16a	Describe the results of the search and selection process, from the number of records identified in the search to the number of studies included in the review, ideally using a flow diagram.	p.7
	16b	Cite studies that might appear to meet the inclusion criteria, but which were excluded, and explain why they were excluded.	p.7
Study characteristics	17	Cite each included study and present its characteristics.	p.8, Table1
Risk of bias in studies	18	Present assessments of risk of bias for each included study.	p.10
Results of individual studies	19	For all outcomes, present, for each study: (a) summary statistics for each group (where appropriate) and (b) an effect estimate and its precision (e.g. confidence/credible interval), ideally using structured tables or plots.	p.8,9,10
Results of syntheses	20a	For each synthesis, briefly summarise the characteristics and risk of bias among contributing studies.	p.1
	20b	Present results of all statistical syntheses conducted. If meta-analysis was done, present for each the summary estimate and its precision (e.g. confidence/credible interval) and measures of statistical heterogeneity. If comparing groups, describe the direction of the effect.	p.8-9
	20c	Present results of all investigations of possible causes of heterogeneity among study results.	p.8-9
	20d	Present results of all sensitivity analyses conducted to assess the robustness of the synthesized results.	p.8-9
Reporting biases	21	Present assessments of risk of bias due to missing results (arising from reporting biases) for each synthesis assessed.	p.10, Supplementary Figure 9”
Certainty of evidence	22	Present assessments of certainty (or confidence) in the body of evidence for each outcome assessed.	Figure 2-3, Supplementary Figure 3-5
**DISCUSSION**			
Discussion	23a	Provide a general interpretation of the results in the context of other evidence.	p.10-14
	23b	Discuss any limitations of the evidence included in the review.	p.12
	23c	Discuss any limitations of the review processes used.	p.12
	23d	Discuss implications of the results for practice, policy, and future research.	p.12-14
**OTHER INFORMATION**			
Registration and protocol	24a	Provide registration information for the review, including register name and registration number, or state that the review was not registered.	Not registered
	24b	Indicate where the review protocol can be accessed, or state that a protocol was not prepared.	No protocol prepared
	24c	Describe and explain any amendments to information provided at registration or in the protocol.	Not applicable
Support	25	Describe sources of financial or non-financial support for the review, and the role of the funders or sponsors in the review.	p.1
Competing interests	26	Declare any competing interests of review authors.	p.1
Availability of	27	Report which of the following are publicly available and where they can be found: template data collection forms; data extracted from included	p.1

Section and Topic	Item #	Checklist item	Location where item is reported
data, code and other materials		studies; data used for all analyses; analytic code; any other materials used in the review.	

From: Page MJ, McKenzie JE, Bossuyt PM, Boutron I, Hoffmann TC, Mulrow CD, et al. The PRISMA 2020 statement: an updated guideline for reporting systematic reviews. BMJ 2021;372:n71. doi: 10.1136/bmj.n71

For Abstracts Checklist

Section and Topic	Item #	Checklist item	Reported (Yes/No)
**TITLE**			
Title	1	Identify the report as a systematic review.	Yes
**BACKGROUND**			
Objectives	2	Provide an explicit statement of the main objective(s) or question(s) the review addresses.	Yes
**METHODS**			
Eligibility criteria	3	Specify the inclusion and exclusion criteria for the review.	Yes
Information sources	4	Specify the information sources (e.g. databases, registers) used to identify studies and the date when each was last searched.	Yes
Risk of bias	5	Specify the methods used to assess risk of bias in the included studies.	Yes
Synthesis of results	6	Specify the methods used to present and synthesise results.	Yes
**RESULTS**			
Included studies	7	Give the total number of included studies and participants and summarise relevant characteristics of studies.	Yes
Synthesis of results	8	Present results for main outcomes, preferably indicating the number of included studies and participants for each. If meta-analysis was done, report the summary estimate and confidence/credible interval. If comparing groups, indicate the direction of the effect (i.e. which group is favoured).	Yes
**DISCUSSION**			
Limitations of evidence	9	Provide a brief summary of the limitations of the evidence included in the review (e.g. study risk of bias, inconsistency and imprecision).	Yes
Interpretation	10	Provide a general interpretation of the results and important implications.	Yes
**OTHER**			
Funding	11	Specify the primary source of funding for the review.	Yes
Registration	12	Provide the register name and registration number.	Not applicable

Source: *Page MJ, McKenzie JE, Bossuyt PM* et al. The PRISMA 2020 statement: an updated guideline for reporting systematic reviews. BMJ 2021; 372: n71. doi: 10.1136/bmj.n71. This work is licensed under CC BY 4.0. To view a copy of this license, visit https://creativecommons.org/licenses/by/4.0/

Supplementary Table 2 Full electronic search strategy (as of October 14, 2024).
Detailed search strategies for each database searched, including Ovid-MEDLINE, Ovid-EMBASE, and the Cochrane Central Register of Controlled Trials. The table outlines search terms, Boolean operators, truncation symbols, controlled vocabulary (e.g., MeSH, Emtree), and the number of results retrieved per query. This strategy was used to identify eligible studies for inclusion in the systematic review.
Abbreviations: MeSH, Medical Subject Headings.

Ovid-MEDLINE (1946 to 2024 October 16)

Research	Query	Items found
1	confocal microscopy.mp. or exp confocal microscopy/	89,137
2	endoscopy.mp. or exp Endoscopy/	465,381
3	#1 AND #2	922
4	(confocal adj3 microscop*).mp.	109,651
5	(confocal adj3 endomicroscop*).mp.	1,462
6	(confocal adj3 microendoscop*).mp.	63
7	OR/#3-6	110,277
8	Barrett Esophagus.mp. or exp Barrett esophagus/	9,305
9	Esophageal Neoplasms.mp. or exp esophagus tumor/	62,033
10	esophageal adenocarcinoma.mp.	5,537
11	Stomach Neoplasms.mp. or exp stomach tumor/	116,405
12	gastric adenocarcinoma.mp.	9,141
13	((esophag* or gastr*) and (lesion or dysplas*)).mp.	31,894
14	OR/#8-13	197,355
15	#7 AND #14	660

Ovid-Embase (1947 to 2024 October 16)

Research	Query	Items found
1	confocal microscopy.mp. or exp confocal microscopy/	143,399
2	endoscopy.mp. or exp Endoscopy/	865,192
3	#1 AND #2	1,963
4	(confocal adj3 microscop*).mp.	190,188
5	(confocal adj3 endomicroscop*).mp.	2,810
6	(confocal adj3 microendoscop*).mp.	91
7	OR/#3-6	190,404
8	Barrett Esophagus.mp. or exp Barrett esophagus/	20,599
9	Esophageal Neoplasms.mp. or exp esophagus tumor/	113,045
10	esophageal adenocarcinoma.mp.	18,186
11	Stomach Neoplasms.mp. or exp stomach tumor/	199,185
12	gastric adenocarcinoma.mp.	13,540
13	((esophag* or gastr*) and (lesion or dysplas*)).mp.	76,587
14	OR/#8-13	350,832
15	#7 AND #14	2,106

EBM Reviews - Cochrane Central Register of Controlled Trials (September 2024)

Research	Query	Items found
1	confocal microscopy.mp. or exp confocal microscopy/	823
2	endoscopy.mp. or exp Endoscopy/	36,815
3	#1 AND #2	41
4	(confocal adj3 microscop*).mp.	1,060
5	(confocal adj3 endomicroscop*).mp.	138
6	(confocal adj3 microendoscop*).mp.	3
7	OR/#3-6	1,116
8	Barrett Esophagus.mp. or exp Barrett esophagus/	602
9	Esophageal Neoplasms.mp. or exp esophagus tumor/	2,609
10	esophageal adenocarcinoma.mp.	469
11	Stomach Neoplasms.mp. or exp stomach tumor/	4,175
12	gastric adenocarcinoma.mp.	843
13	((esophag* or gastr*) and (lesion or dysplas*)).mp.	2,804
14	OR/#8-13	9,673
15	#7 AND #14	57

Supplementary Table 3 Diagnostic accuracy summary for probe-based confocal laser endomicroscopy and white-light endoscopy.

Study	Modality	TP	FP	FN	TN	Sensitivity	Specificity
Kobayashi (2016)	pCLE	13	2	5	10	0.72	0.83
Lim (2013)	pCLE	60	9	6	50	0.91	0.85
Sharma (2011)	pCLE	82	92	38	662	0.68	0.88
Kobayashi (2016)	WLE	11	3	7	9	0.61	0.75
Lim (2013)	WLE	25	3	41	56	0.38	0.95
Sharma (2011)	WLE	41	55	79	699	0.34	0.93

True positive (TP), false positive (FP), false negative (FN), and true negative (TN) counts with derived sensitivity and specificity values across selected comparative studies of probe-based confocal laser endomicroscopy and white-light endoscopy.
pCLE, probe-based confocal laser endomicroscopy.

Supplementary Table 4 Additional detection and diagnostic yield using pCLE.

Study focus	Findings
Barrett's esophagus	pCLE detected lesions not seen with conventional endoscopy (6/8 studies)
Head and neck cancer	Additional lesion detection via pCLE (one study)
Gastric cancer (lesion targeting)	pCLE-guided biopsies identified more undifferentiated cancer cells (one study)
Gastric cancer (tissue yield)	Higher rate of malignant tissue inclusion than WLE or ME-NBI (two studies)
Gastric cancer (diagnostic Yield)	Higher yield than WLE or pretreatment biopsy; greater than FICE alone (one study)

FICE, flexible imaging color enhancement; ME-NBI, magnifying endoscopy with narrow-band imaging; pCLE, probe-based confocal laser endomicroscopy; WLE, white-light endoscopy.

Supplementary Table 5 Study-level details of the clinical impact of pCLE on management change and biopsy reduction.

Study (year)	Outcome assessed	Clinical setting	Comparator modality	Unit of analysis (denominator)	Direction of management effect
Caillol (2017)	Management change	Barrett's esophagus patients presenting with subtle mucosal irregularities (without macroscopic lesions)	Standard pre-resection biopsy	Per-patient (n = 13)	Altered real-time therapeutic management in 69.2% (9/13) of patients by either prompting endoscopic resection for suspected higher-grade disease or avoiding unnecessary resection/RFA.
**Zuo (2017)**	Biopsy reduction	Patients with suspected gastric precancerous lesions (atrophic gastritis, GIM, or GIN)	FICE alone	Per-patient (n = 238)	FICE-guided pCLE significantly reduced the mean number of biopsies per patient by 48.5% compared with FICE alone (3.5 vs. 6.8; $p < 0.001$).
Guo (2015)	Biopsy reduction	Early esophageal squamous neoplasia	Standard WLE/I-Scan	Per-lesion (n = 91 suspected lesions)	The high negative predictive value of pCLE allowed targeted biopsy to be safely avoided in 56.0% (51/91) of non-neoplastic appearing lesions.
Sharma (2011)	Biopsy reduction	Barrett's esophagus surveillance	HD-WLE + NBI	Per-patient (n = 101)	The adjunctive use of pCLE allowed 39% of patients to completely avoid unnecessary random 4-

	quadrant biopsies without missing any cases of HGD or early cancer.

ER, endoscopic resection; FICE, flexible spectral imaging color enhancement; GIM, gastric intestinal metaplasia; GIN, gastric intraepithelial neoplasia; HD-WLE, high-definition white-light endoscopy; HGD, high-grade dysplasia; NBI, narrow-band imaging; NPV, negative predictive value; pCLE, probe-based confocal laser endomicroscopy; RFA, radiofrequency ablation.

Supplementary Figures

Supplementary Fig. 1 Risk of bias assessment and Applicability Summary (QUADAS-2). Graphical summary of risk of bias and applicability concerns across included studies, assessed using the QUADAS-2 tool. Ratings are presented for each domain: patient selection, index test, reference standard, and flow and timing. Most studies showed low risk of bias; unclear or high risk was noted in the index test domain in a subset of studies. QUADAS-2, Quality Assessment of Diagnostic Accuracy Studies-2.

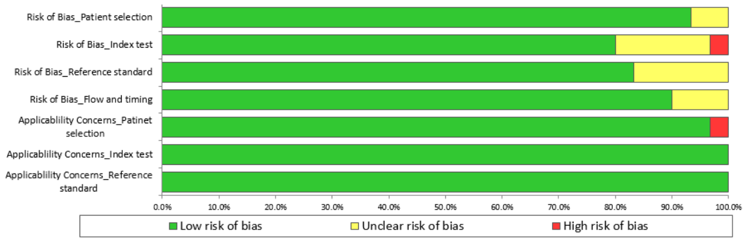

Author (year)	Risk of Bias				Applicablility Concerns		
	Patient selection	Index test	Reference standard	Flow and timing	Patinet selection	Index test	Reference standard
Pilonis(2023)	!	✓	✓	!	✓	✓	✓
Seerani(2023)	✓	✓	✓	✓	✓	✓	✓
Kim(2022)	✓	!	!	✓	✓	✓	✓
Pang(2022)	✓	✓	✓	✓	✗	✓	✓
Vithayathil(2022)	✓	!	✓	✓	✓	✓	✓
Chu(2021)	✓	!	!	✓	✓	✓	✓
Schueler(2021)	✓	✓	✓	✓	✓	✓	✓
Kollar(2020)	✓	✓	✓	✓	✓	✓	✓
Krajciova(2020)	✓	✓	✓	✓	✓	✓	✓
Prueksapanich(2019)	✓	✓	✓	!	✓	✓	✓
Richardson(2019)	✓	✓	✓	✓	✓	✓	✓
Chen(2018)	!	!	!	✓	✓	✓	✓
Horiguchi(2018)	✓	✓	✓	✓	✓	✓	✓
Shah(2018)	✓	✓	✓	✓	✓	✓	✓
Caillol(2017)	✓	✓	!	!	✓	✓	✓
Safatle-Ribeiro(2017)	✓	✓	✓	✓	✓	✓	✓
Zuo(2017)	✓	✓	✓	✓	✓	✓	✓
Kobayashi(2016)	✓	✓	✓	✓	✓	✓	✓
Li(2016)	✓	✓	✓	✓	✓	✓	✓
di Pietro(2015)	✓	✗	!	✓	✓	✓	✓
Gong(2015)	✓	✓	✓	✓	✓	✓	✓
Guo(2015)	✓	✓	✓	✓	✓	✓	✓
Prueksapanich(2015)	✓	!	✓	✓	✓	✓	✓
Bertani(2013)	✓	✓	✓	✓	✓	✓	✓
Bok(2013)	✓	✓	✓	✓	✓	✓	✓
Lim(2013)	✓	✓	✓	✓	✓	✓	✓
Pittayanon(2013)	✓	✓	✓	✓	✓	✓	✓
Sharma(2011)	✓	✓	✓	✓	✓	✓	✓
Bajbouj(2010)	✓	✓	✓	✓	✓	✓	✓
Pohl(2008)	✓	✓	✓	✓	✓	✓	✓

Supplementary Fig. 2 Broader esophageal analyses across all eligible esophageal-target studies. **a** Forest plot of pooled sensitivity and specificity. **b** SROC curve. This analysis includes studies targeting dysplasia, intestinal metaplasia, or neoplasia and complements the neoplasia-focused primary analysis in **Fig. 2**.
pCLE, probe-based confocal laser endomicroscopy; SROC, summary receiver operating characteristic.

(A)

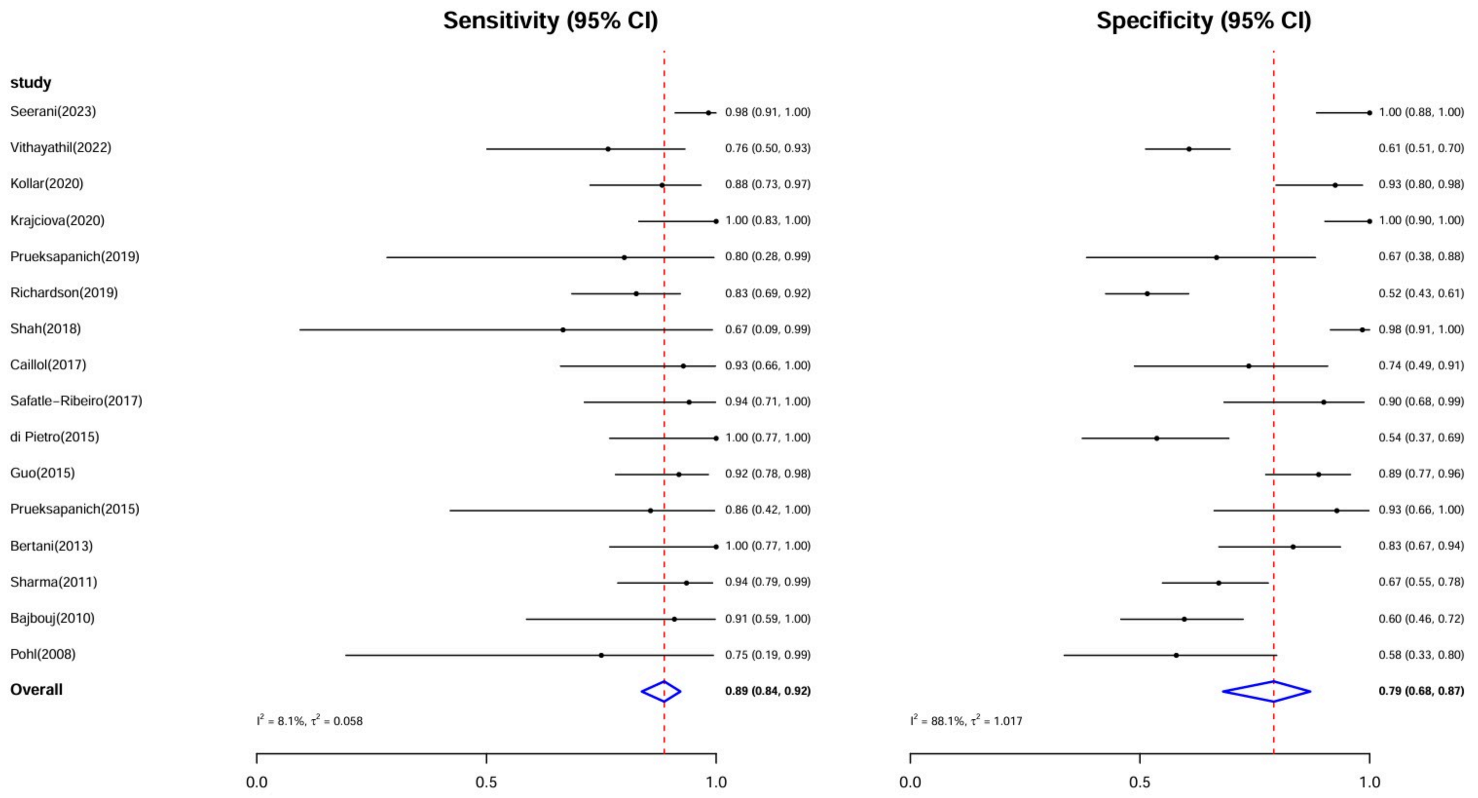

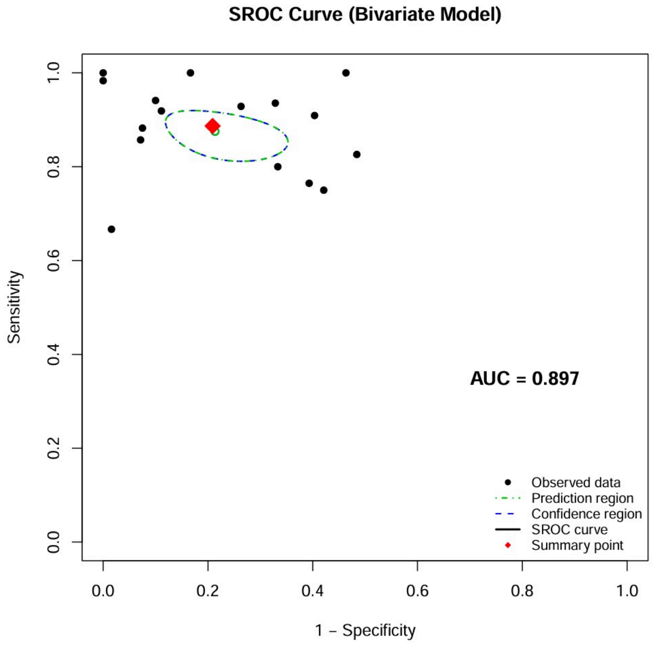

(B)

Supplementary Fig. 3 Broader gastric analyses across all eligible gastric-target studies. **a** Forest plot of pooled sensitivity and specificity. **b** SROC curve. This analysis includes metaplastic and lower-grade precursor lesions and complements the neoplasia-focused primary analysis in **Fig. 2**.
pCLE, probe-based confocal laser endomicroscopy; SROC, summary receiver operating characteristic.

(A)

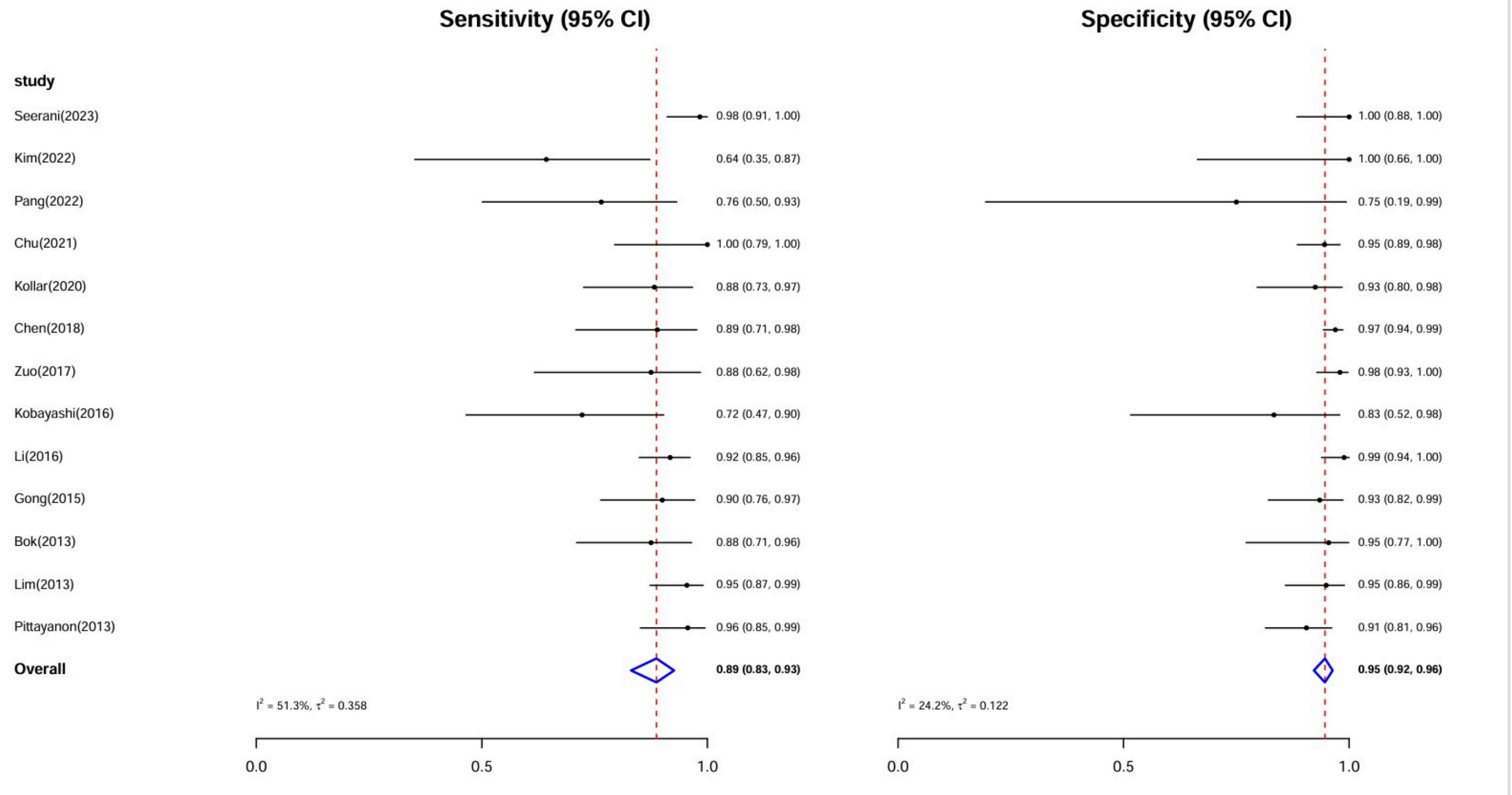

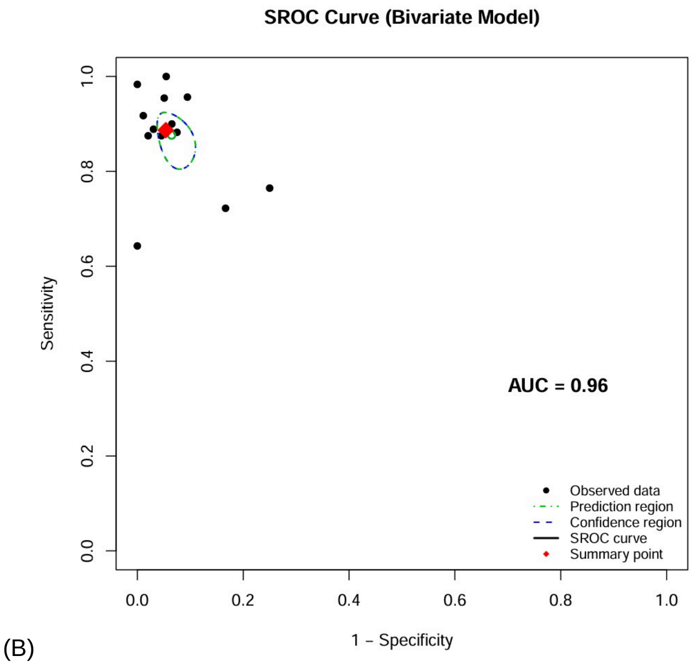

(B)

Supplementary Fig. 4 Sensitivity analyses restricted to studies reporting per-patient esophageal data. **a** Forest plot of pooled sensitivity and specificity. **b** SROC curve. This analysis was performed to address heterogeneity in the unit of analysis across included studies.
SROC, summary receiver operating characteristic.

(A)

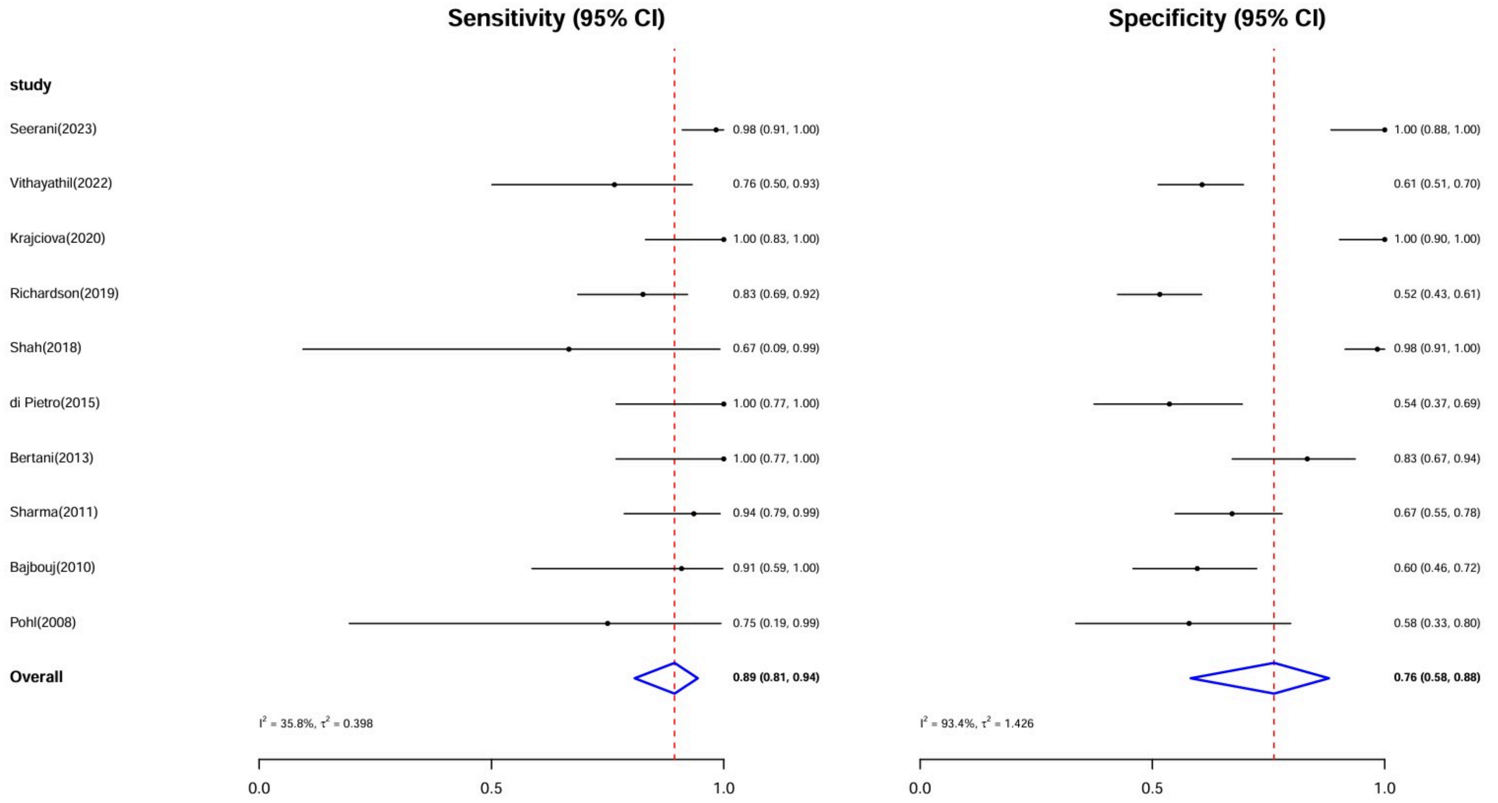

(B)

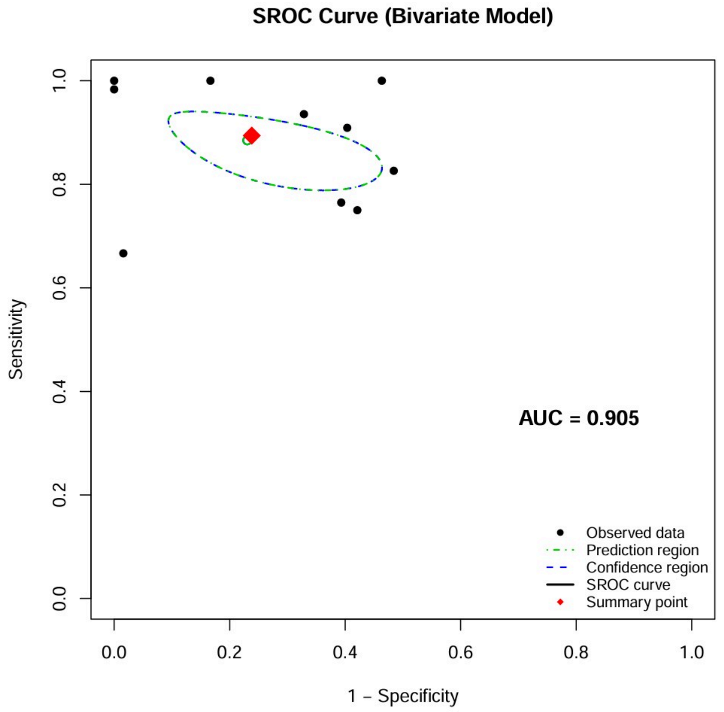

Supplementary Fig. 5 Sensitivity analyses restricted to studies reporting per-patient gastric data. **a** Forest plot of pooled sensitivity and specificity. **b** SROC curve. This analysis was performed to assess the effect of mixed analytic units in the primary synthesis.
SROC, summary receiver operating characteristic.

(A)

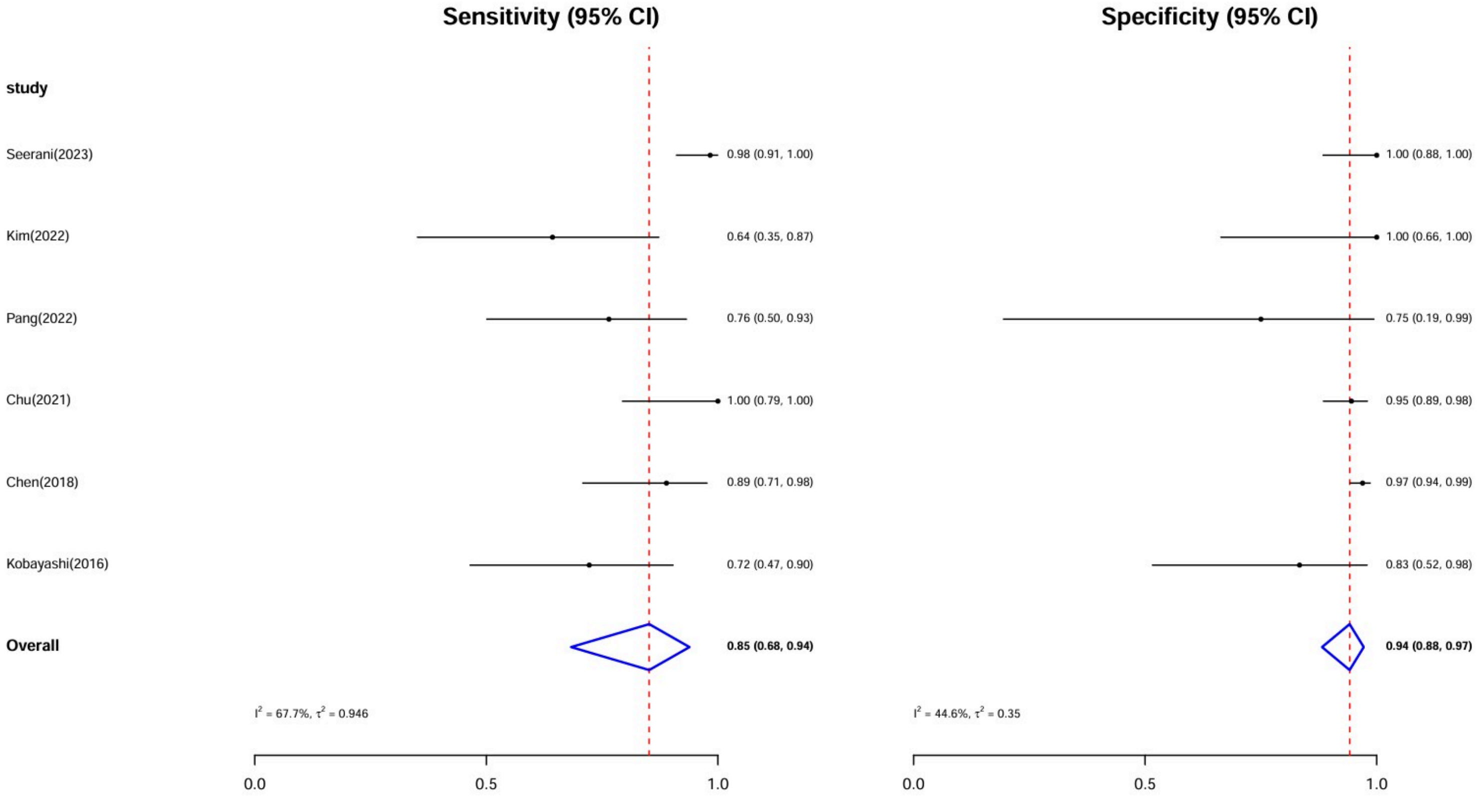

(B)

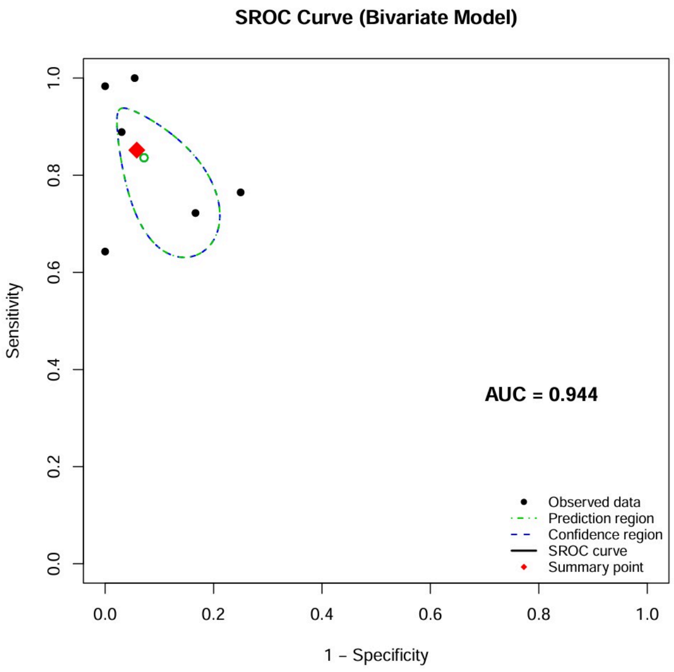

Supplementary Fig. 6 Sensitivity analyses restricted to studies reporting per-patient data for high-grade or frankly neoplastic lesions. **a** Forest plot of pooled sensitivity and specificity. **b** SROC curve.
SROC, summary receiver operating characteristic.

(A)

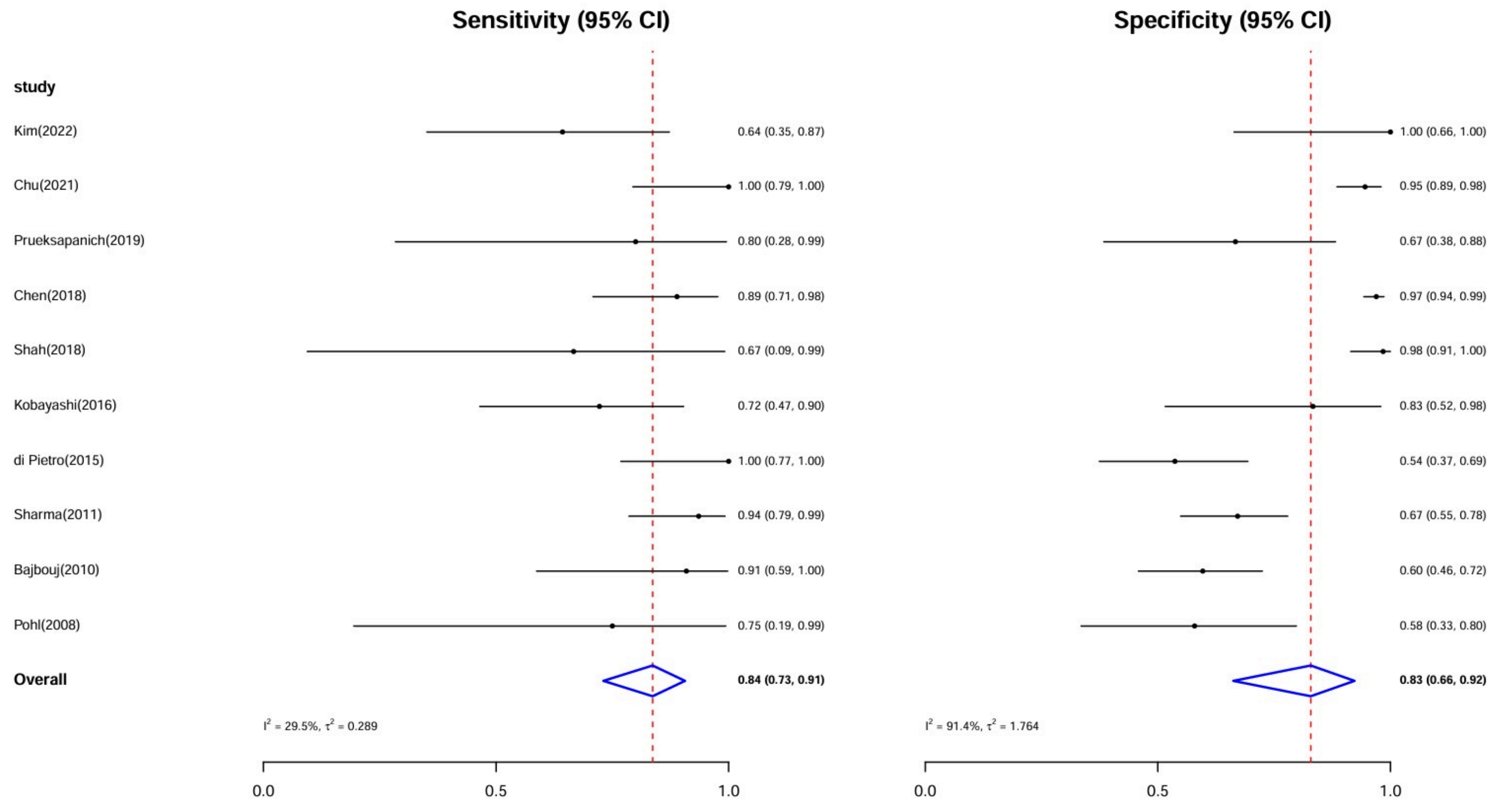

(B)

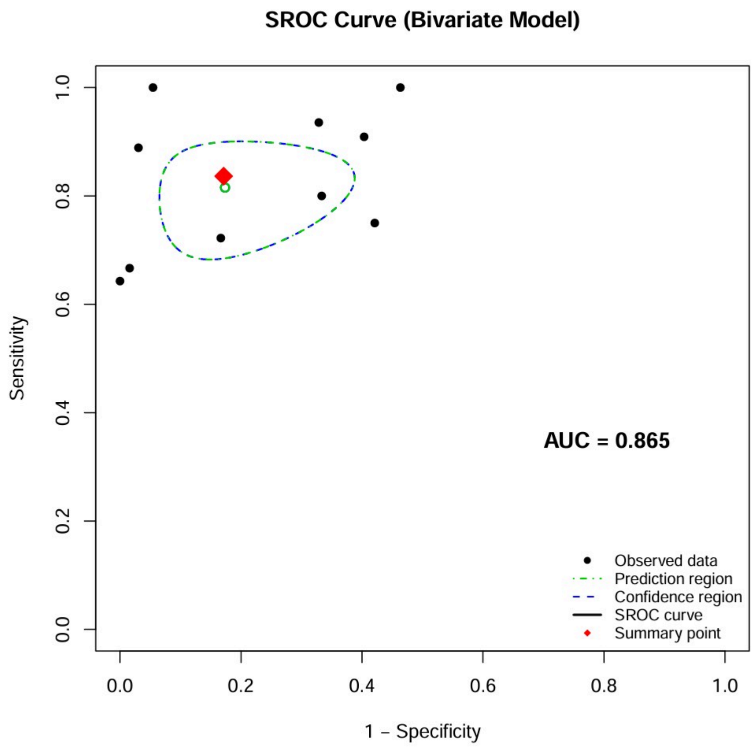

Supplementary Fig. 7 Sensitivity analyses restricted to studies reporting per-patient data for lower-grade or metaplastic lesions, including LGD and GIM. **a** Forest plot of pooled sensitivity and specificity. **b** SROC curve. Gastrointestinal metaplasia; LGD, low-grade dysplasia; SROC, summary receiver operating characteristic.

(A)

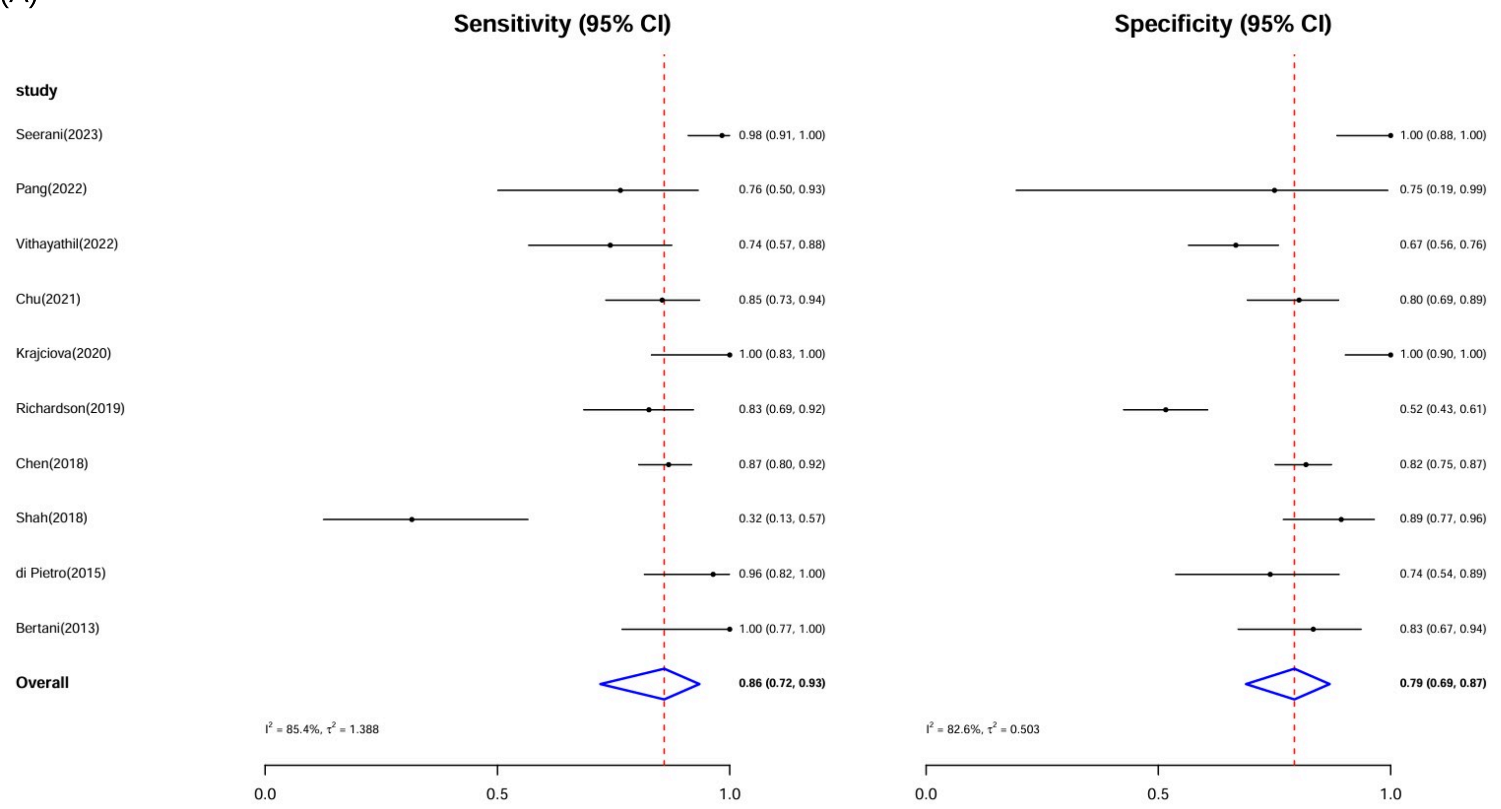

(B)

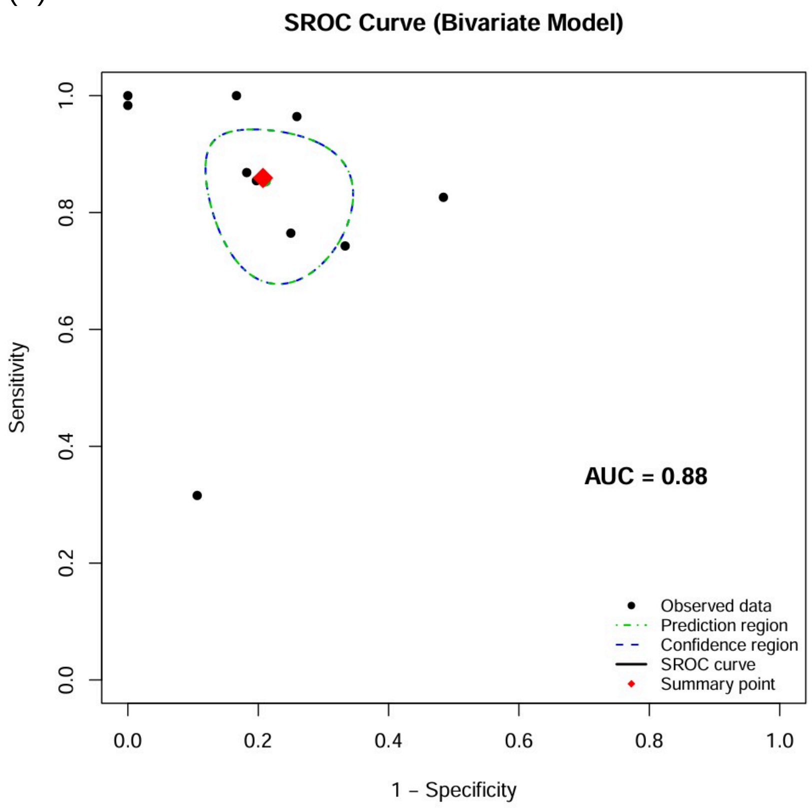

Supplementary Fig. 8 Barrett's esophagus-focused subgroup analyses. **a** Forest plot of pooled sensitivity and specificity. **b** SROC curve. These analyses contextualize the Barrett-focused studies that also reported incremental lesion detection and management modification.
pCLE, probe-based confocal laser endomicroscopy.

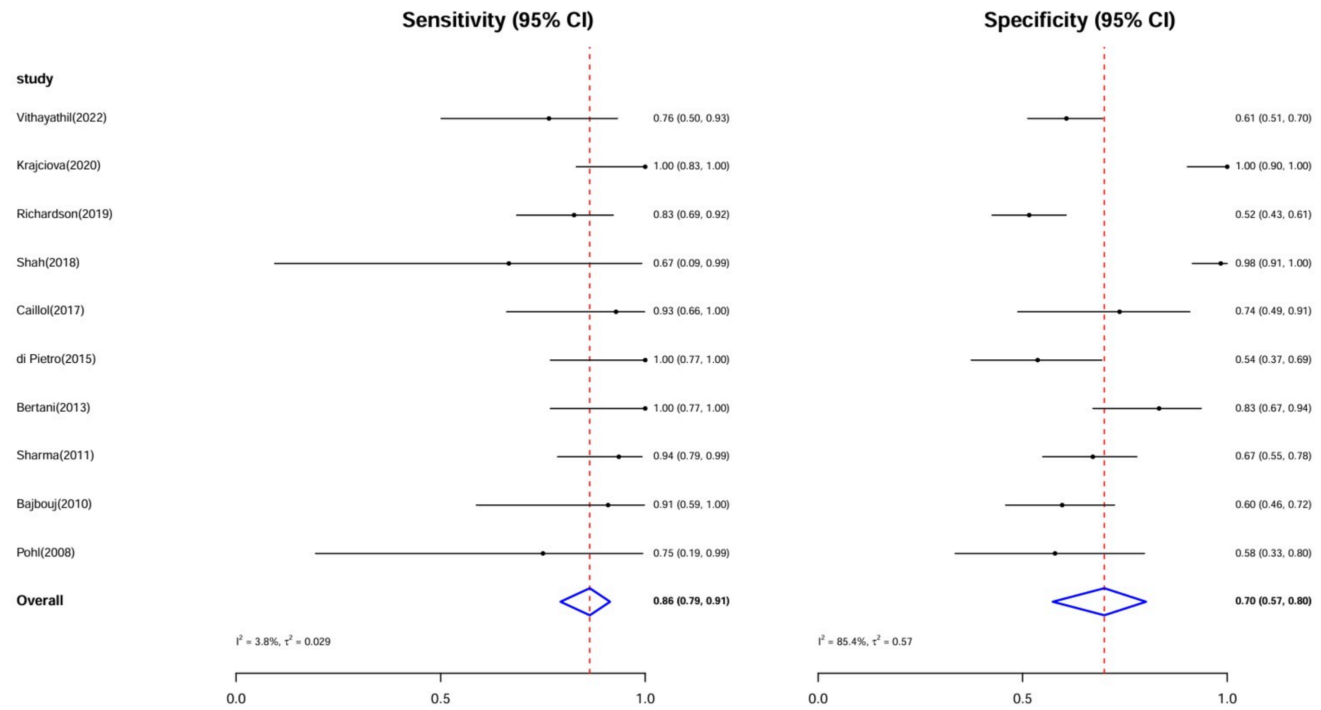

(A)

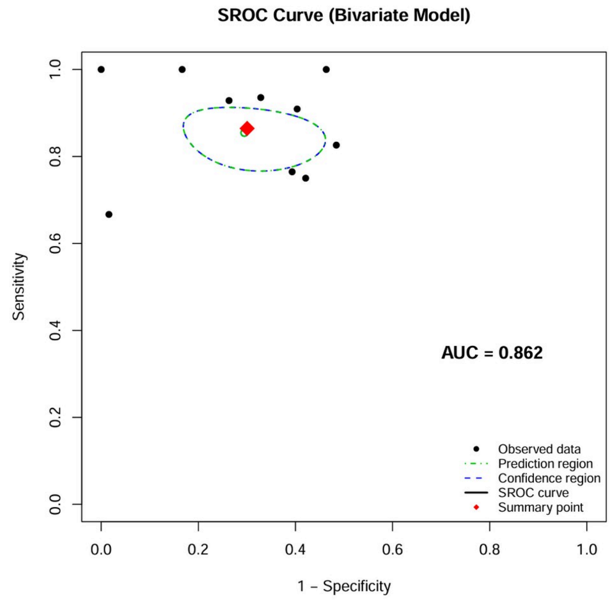

(B)

Supplementary Fig. 9 Funnel plots for Publication Bias Assessment.
a Funnel plot for studies assessing pCLE in esophageal neoplasia. Deeks' test did not provide strong statistical evidence of funnel plot asymmetry (P = 0.12); however, given the limited number of studies and the low power of asymmetry tests in small meta-analyses, publication bias or small-study effects cannot be excluded. **b** Funnel plot for studies on gastric neoplasia. The observed asymmetry and statistically significant Deeks' test (P = 0.01) suggest potential small-study effects and publication bias.
pCLE, probe-based confocal laser endomicroscopy.

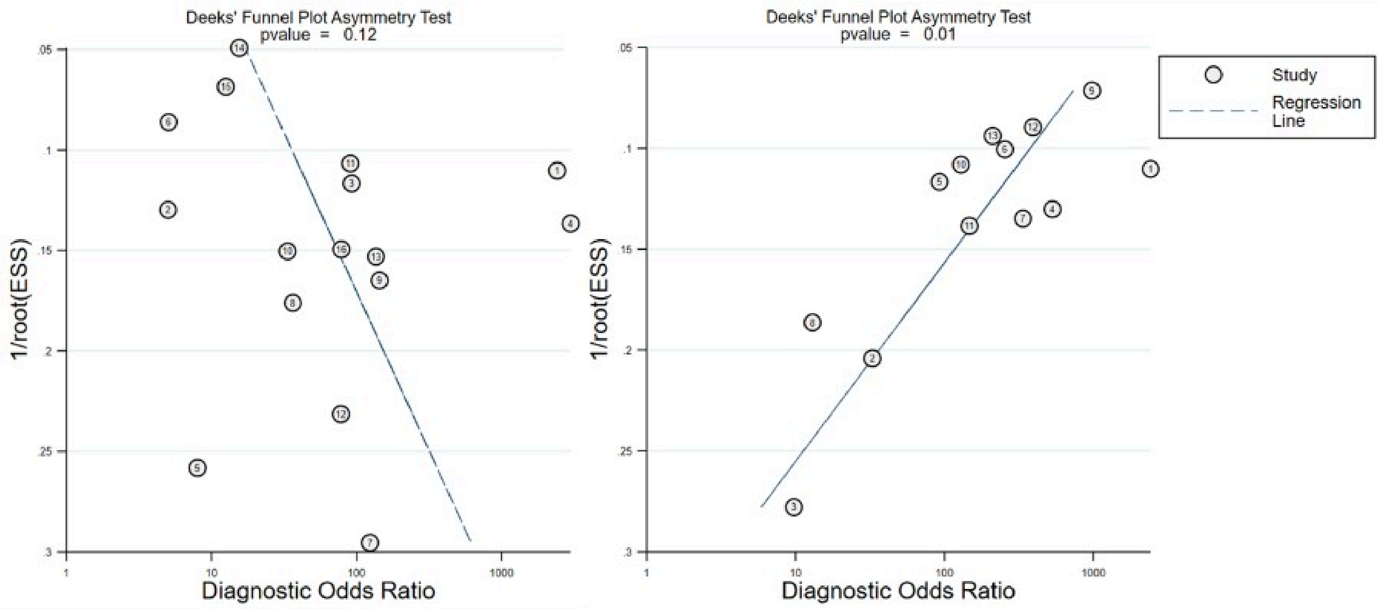

Supplement: Supplementary file 1 — Supplementary Material [file 10-1055-a-2863-1407_28644724.pdf]
